# Two-dimensional material integrated micro-nano fiber, the new opportunity in all-optical signal processing

**DOI:** 10.1515/nanoph-2023-0223

**Published:** 2023-05-16

**Authors:** Xinyu Wang, Wanzhuo Ma, Yanwei Fu, Xianzhu Liu, Zonghui Tao, Yansong Song, Keyan Dong, Huilin Jiang

**Affiliations:** College of Opto-Electronic Engineering, Changchun University of Science and Technology, Changchun 130022, China

**Keywords:** 2D materials, all-optical signal processing, microfiber

## Abstract

With the development of all-optical networks, all-optical devices have become a research hotspot in recent years. Two-dimensional materials, represented by graphene and black phosphorus, have attracted great interest in the scientific community due to their excellent optical, electrical, magnetic, and mechanical properties. Bridging the gap between fiber optics and nanotechnology, microfibers can interact with light and matter at the micro or even nanoscale. By combining two-dimensional materials with microfibers, composite waveguides can be formed. They have the advantages of high nonlinear effect, all-fiber structure, and high damage threshold, etc. The composite waveguide can be directly applied to optical fiber communication systems, and plays an important role in the field of all-optical signal processing with a huge application prospect. In this review, the properties of typical 2D materials are first introduced. Next, the preparation methods of the relevant equipments are introduced and compared. Then, the all-optical signal processing technology based on 2D material-integrated microfiber composite waveguide is reviewed. The latest developments of all-optical modulators, all-optical wavelength converters, all-optical logic gates and all-optical thresholding devices are presented. Finally, the challenges and opportunities for the future development of 2D materials-integrated microfiber optoelectronic devices are summarized.

## 1 Introduction

Compared with the traditional “electrical network”, the all-optical network has the advantages of simple structure, high throughput capacity, openness, reconfigurability, low cost, and so on. In the all-optical network, the transmission, exchange, and amplification of information do not require photoelectric and electro-optical conversion, so the communication speed is no longer limited by the response speed of electronic devices, effectively solving the “electronic bottleneck” problem existing in the electrical network. Traditional all-optical modulators, wavelength converters, analogue-to-digital converters, etc. They mostly use optical devices with high nonlinearity as the working medium, such as semiconductor optical amplifiers, semiconductor lasers, nonlinear optical fibers, and photonic crystal fibers, and so on. However, the above bulk structures increase the complexity and cost of the optical communication system. Therefore, the exploration of all-optical signal processing devices with broadband optical response, high efficiency, and high integration has always been the focus of researchers.

The excellent nonlinear effect and strong light–matter interaction of two-dimensional materials can compensate for the shortcomings of silicon-based semiconductor materials, and their preparation is not difficult, opening a new possibility for the realization of highly integrated all-optical signal processing. Since the isolation of a single layer of graphite by a mechanical exfoliation method by an experimental group at the University of Manchester in 2004 [1], these two-dimensional materials have attracted widespread attention in the scientific community. By studying the unique energy band structure of graphene, scientists have discovered many excellent properties of graphene, such as excellent electron mobility, thermal conductivity, and excellent intrinsic tensile strength, wavelength-independent saturable absorption, ultrafast nonlinear optical response, and nonlinear optical properties [2–4]. It has great potential in research areas such as optics and electricity [5–8]. Since then, a number of 2D materials have been successively isolated, such as 2D transition metal sulfides [9–12], topological insulators [13–16], black phosphorus [16–18], MXene [19, 20], which exhibit peculiar electronic and optical properties that are fundamentally different from those of bulk materials [21]. For example, the highly tunable bandgap provides an extremely wide range of optical responses and is easily integrated with photonic structures such as fiber [22], and chips [23, 24], offering more possibilities for photonic devices, providing new opportunities and developments for nanoelectronics and photonics [2, 21, 25]. Two-dimensional materials represented by graphene have been used in many photonic and optoelectronic devices, such as generating ultrashort pulses [22, 26, 27], terahertz devices [28], etc. Recently, a new type of two-dimensional material, MBenes, has attracted the attention of researchers. A large number of studies and theories have shown that MBenes has exciting properties [29], and its potential in the field of photonics is yet to be discovered.

A challenging problem is how to achieve high-efficiency, low-loss interactions of 2D materials with light waves. In 2003, Tong first proposed an ultra-low-loss microfiber (MF) with a subwavelength diameter in Nature [30], which opened a new door for high-efficiency coupling between two-dimensional materials and light waves. As the most basic optical transmission unit in microfiber devices, wavelength or sub-wavelength-scale optical waveguides have become a research hotspot in current photonics. Compared with conventional fibers, microfibers guide light beams by total internal reflection at the material-air interface. At present, the widely reported micro-nano optical waveguides mainly include silicon-based waveguides, metal nanowires, photonic crystal waveguides, and microfibers, etc. Microfiber is a special fiber with a diameter of micrometers or nanometers, made from standard fiber through a series of processes, which has a natural pigtail for easy integration with conventional optical systems. Microfiber has excellent properties such as large-scale evanescent field transmission, large waveguide dispersion, and strong optical field confinement [31, 32], etc. It has great application value in resonance, sensing [33, 34], nonlinear optics [35, 36] and other fields. The two-dimensional materials can be coated onto the microfiber by optical deposition method, and the interaction between light and the material can be enhanced by exploiting the large evanescent field properties of microfiber.

The evanescent field interaction along the microfiber can greatly increase the interaction length between the two-dimensional materials and the propagating light. At the same time, the microfiber itself also has a high degree of optical nonlinearity. Two-dimensional materials-integrated microfiber composite waveguide has been effectively applied in many fields. Passive Q-switching and mode-locking based on saturable absorbing materials are the two main mechanisms for realizing pulsed lasers [37–40]. Many researchers have applied graphene to saturable absorber due to its broadband absorption properties from visible to infrared, good modulation depth, low threshold for saturable absorption, the resistance to optical damage, graphene-based ultrafast lasers have developed rapidly in recent years [2, 41]. Two-dimensional materials such as topological insulators and black phosphorus have also been shown to be saturable absorbers, and two-dimensional materials-integrated microfiber composite waveguides are beneficial for ultrafast fiber laser research [42–48]. At the same time, taking advantage of the excellent nonlinear effects of these two-dimensional materials, the composite waveguide is also extremely useful in all-optical signal processing, including all-optical modulators, all-optical wavelength converters, and all-optical thresholding devices with a great research value [49–52]. For example, all-optical modulators based on graphene coated microfiber have been successfully demonstrated [53]. Researchers have also successfully developed an antimonene-based all-optical modulator and applied it to an active Q-switched laser [54]. We have noticed that there are some reviews based on 2D materials-decorated microfiber devices, and these reviews have reviewed pulse generation and all-optical devices such as all-optical modulators related to two-dimensional materials [55–58].

This paper mainly reviews the latest progress in the application of two-dimensional materials-integrated microfiber composite waveguides. Section 2 briefly introduces the properties of typical 2D materials. The commonly used fabrication methods of microfibers and the commonly used processing of 2D materials are analyzed and compared in Section 3. In Section 4, the latest advances of composite waveguides in all-optical signal processing technology, such as all-optical modulators, all-optical wavelength converters, and all-optical thresholding devices are introduced. In Section 5, the 2D materials-integrated microfiber composite waveguide are summarized and the opportunities and challenges they will face in the future are briefly analyzed.

## 2 Properties of typical 2D materials

Two-dimensional materials are also known as two-dimensional monolayers or topological materials, and they are usually composed of one or several layers of atoms. With the discovery of graphene, graphene and other 2D materials have attracted the attention of researchers for their unique optical, electrical, and other properties. To date, as the preparation process continues to mature, more and more 2D materials have been discovered and are widely used in experimental research. The advent of 2D materials has ushered in a new era. In this section, we will focus on the fundamental properties of typical 2D materials, including graphene, black phosphorus (BP), MXene, and some recent novel materials will also be briefly introduced.

### 2.1 Graphene

Graphene is one of the most familiar 2D materials, consisting of a single layer of carbon atoms in a hexagonal lattice whose internal carriers can be described as massless Dirac fermions, and whose structure and zero band gap allow for the presence of electron-hole pairs excited by any incident photon resonance [2, 22, 59], [60], [61], [62], [63], allowing the tuning of ultra-wideband, giving graphene additional applications in photonics, electronics, and plasmonic, such as photodetectors, saturable absorbers and modulators [64]. Graphene can interact with light from the ultraviolet to the far infrared [65], and single-layer graphene absorbs 2.3 % of the vertical incident light in the visible and near-infrared spectra. Graphene can also be applied to self-phase modulation and wavelength converter due to its extremely high third-order magnetization-related strong nonlinearity, which is of significant research value [66, 67].

### 2.2 Black phosphorus

Black phosphorus is a single-element layered semiconductor material with a honeycomb structure, the band gap spans from 0.3 eV to 1.5 eV, and the band gap of black phosphorus is related to the number of layers, such as a single layer of black phosphorus band gap of about 1.5 eV, when the number of layers increases to 3, the band gap decreases to 0.8 eV [68, 69]. Monolayer black phosphorus, also known as phosphene, brought BP to the field of photothermal integrated devices in 2020 when researchers prepared a phosphene-assisted MRR-based all-optical modulator [70]. Black phosphorus covers a wide wavelength range from the mid-infrared to the visible spectrum, making it an ideal material for broadband optical applications. Moreover, the strong light–matter interaction and small band gap make black phosphorus one of the candidate materials for nonlinear optics. However, the structure of black phosphorus is not centrosymmetric like graphene and transition metal dichalcogenides (TMDs), leading to high anisotropy in light absorption and photoluminescence of black phosphorus. And because of the instability of black phosphorus in air, it will degrade the electronic and optical properties of BP, so the application of BP to the actual production faces greater difficulties [71].

### 2.3 Transition metal dichalcogenides

TMDs are layered structures that can be represented by the MX_2_ molecular formula, with M denoting transition metal elements (Mo, W, Re) and X denoting (S, Se, Te). TMDs typically have a band gap associated with the layers, and these materials transition from an indirect band gap to a direct band gap as the thickness is reduced from multiple layers to a single layer. TMDs exhibit strong light–matter interactions. Their band gaps range from 1 eV to 2.5 eV and their spectra range from near-infrared to visible, advantageous in achieving broadband absorption and ultra-short pulses [72]. TMDs typically have resonant absorption in the visible light, a property that provides an alternative to graphene-based saturable absorbers (SAs), such as those based on TMDs that have been reported for all-fiber pulsed lasers in the visible range [72–74]. Monolayer MoS_2_-based all-optical modulator has been demonstrated to extend the application of TMDs in infrared optoelectronics, offering the possibility of developing more TMDs-based all-optical modulators [75].

### 2.4 MXene

MXenes include 2D transition metal carbides, hydrocarbons and nitrides, and dozens of different MXenes have been synthesized so far [76]. The difference in structure diversifies the functions of MXenes, making them useful in energy storage, sensing, optoelectronics, and catalysis. The general formula of MXenes is M_n+1_X_n_T_x_, M stands for early transition metals (such as Cr, Mo, Ti, etc.), X stands for carbon or nitrogen, and T_x_ stands for surface terminations (such as hydroxyl, oxygen, or fluorine) [77–80]. Al was selectively extracted from its MAX parent Ti_3_AlC_2_ in aqueous HF solution to obtain Ti_3_C_2_T_x_, which is the first reported MXene [81, 82]. Since then, aqueous acid etching has been widely used to synthesize novel MXene, and it was subsequently found that MXenes could also be synthesized from non-MAX-phase precursors [83, 84] or fabricated using high-temperature etching of the MAX-phase [85]. MXenes operate at wavelengths between ultraviolet and radio waves, with strong energy conversion efficiency, good conductivity, broadband saturation absorption, etc., providing opportunities for ultrafast photonics and all-optical modulation, etc. [58]. For example, in 2019, researchers apply Ti_3_AlC_2_ in wavelength converter with −59 dB conversion efficiency at 10 Ghz modulated signal [86]. As a derivative of MXenes, interest in two-dimensional transition metal borides has continued to grow in recent years. MBenes have good electrical and mechanical properties, and their potential for energy storage and conversion has been proven, attracting the attention of many researchers, but the potential of MBenes for applications in optoelectronic devices still needs to be further explored [29].

## 3 Composite waveguide fabrication

### 3.1 Microfiber fabrication

**Figure 1:**
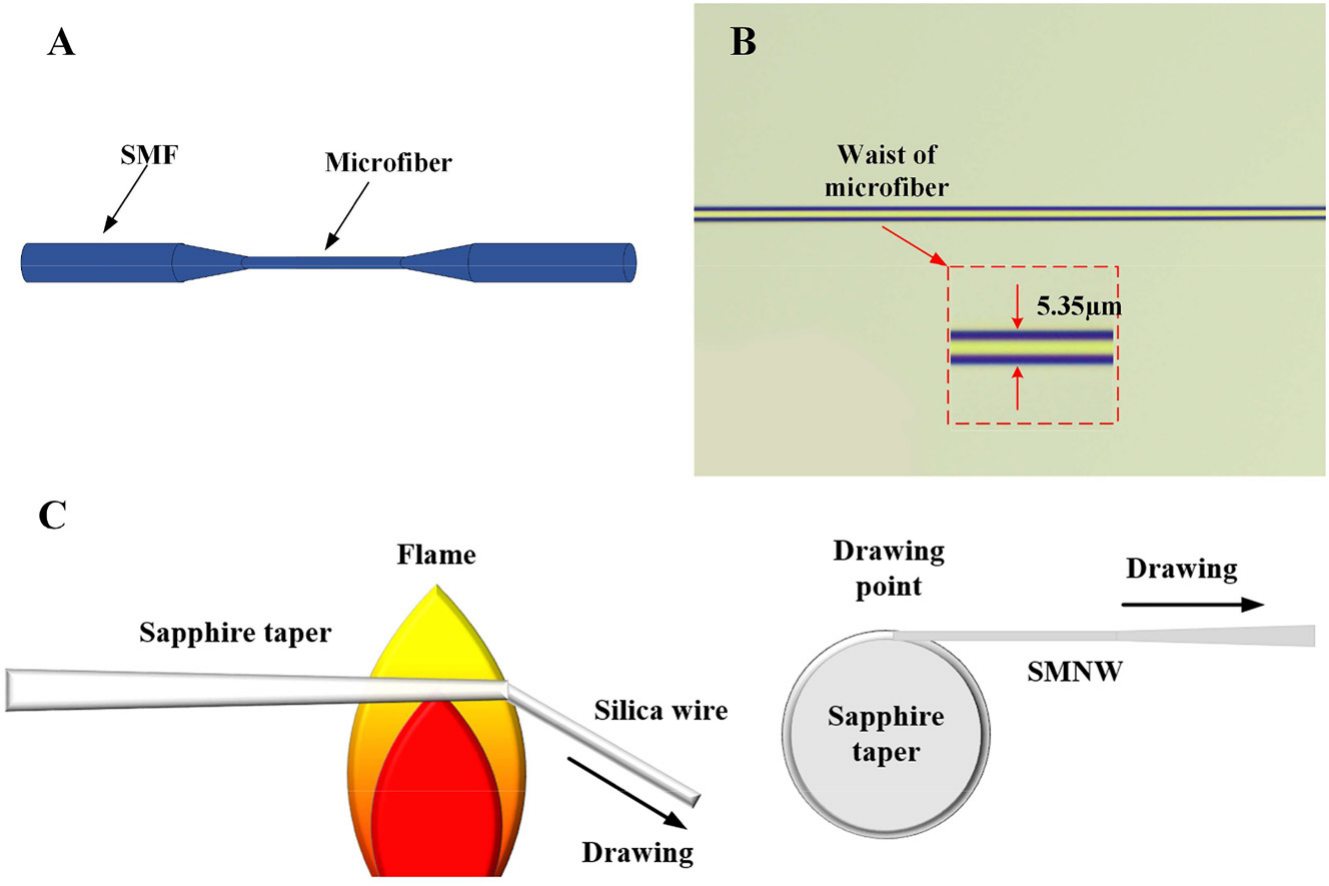
Structure and the two-step drawing process of the microfiber. (A) Microfiber structure. (B) Microscopic image of microfiber. (C) Second step of the two-step drawing process. Reproduced with permission from Ref. [30]. Copyright 2003, Springer Nature.

The structure of the microfiber and the image under the microscope are shown in Figure 1A and B. Research into the production of microfibers began in the 1980s. However, it was limited by the manufacturing process. At the same time, the samples drawn were not only large in diameter, but also had extremely high transmission losses. By 2003, Tong had used a two-step stretching method to obtain silica nanowires that for the first time could achieve low-loss optical transmission at the subwavelength scale [30]. Since then, the research on microfiber has entered a new chapter. At present, the main methods of microfiber fabrication include: direct drawing method [87–89], two-step drawing method (as shown in Figure 1C) [30], flame brushing method [90], and polymer solution drawing, etc. Flame brushing is the most common manufacturing method at present, the microfiber prepared by this method have the characteristics of high fiber quality, high mechanical strength, low-loss and so on.

**Figure 2:**
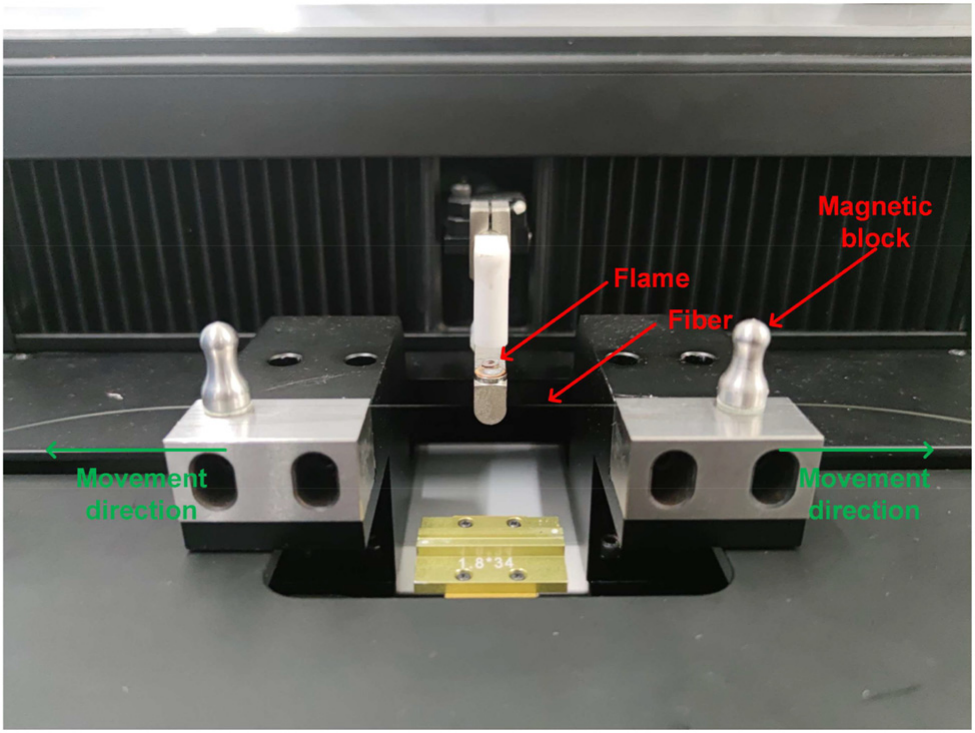
Experimental setup for fabricating tapered fibers by flame-brushing method.

A typical flame-brushing method is shown in Figure 2. The two ends of the exposed SMF (single-mode fiber) are fixed on two moving platforms by magnetic blocks, and the whole process is controlled by a LabVIEW program. While the flame heats the central part of the fiber to soften it, two fiber clamps are stretched at the same speed in both directions under the control of the computer, and the diameter of the tapered fiber can be controlled by the computer. During the fabrication process, a light source is injected into the tapered fiber and the transmission loss of the fiber is monitored by a power meter, which allows the transmission loss of the micro-nano fiber to be controlled within reasonable limits.

The transmission loss of the microfiber is mainly related to the surface roughness and diameter uniformity of the fiber. Air turbulence also indirectly affects the quality of the microfiber. By changing the flame purification, stabilizing the flame position, and manufacturing in a closed environment, researchers have produced microfibers with losses below 0.001 dB/mm [91]. However, the length of the microfiber produced based on these methods is short, and the waist length of the microfiber determines the interaction distance between light and substance, so the production of long-distance microfiber gradually attracting the attention of researchers. In 2019, Lee proposed a method to fabricate ultra-long microfibers, including three steps of traditional flame-brushing and pulling, recalibration, and one-directional pulling [92]. Using this method, two strands of microfibers are fabricated, having 0.82 µm/1.6 µm diameters, 220 mm/500 mm uniform lengths with <55 nm/66 nm diameter variances, and high transmittances of 90.2 %/91.5 %. The previous reported results of microfiber fabrication by flame brushing method are shown in Table 1.

**Table 1:** The previously reported of MNF fabrication by flame brushing method.

Year	*L* (mm)	*D* (µm)	*T* (%)	Ref.
1992	5	43	NA	[93]
2000	90	2	95.5	[35]
2003	4	0.26	20.9	[30]
2013	150	0.9	75	[94]
2014	100	1.2	97.7	[95]
2017	300	1.3	97	[96]
2018	250	1	97	[97]
2019	217	0.82	91.5	[92]
2019	500	1.6	90.2	[92]

### 3.2 Two-dimensional materials fabrication

**Figure 3:**
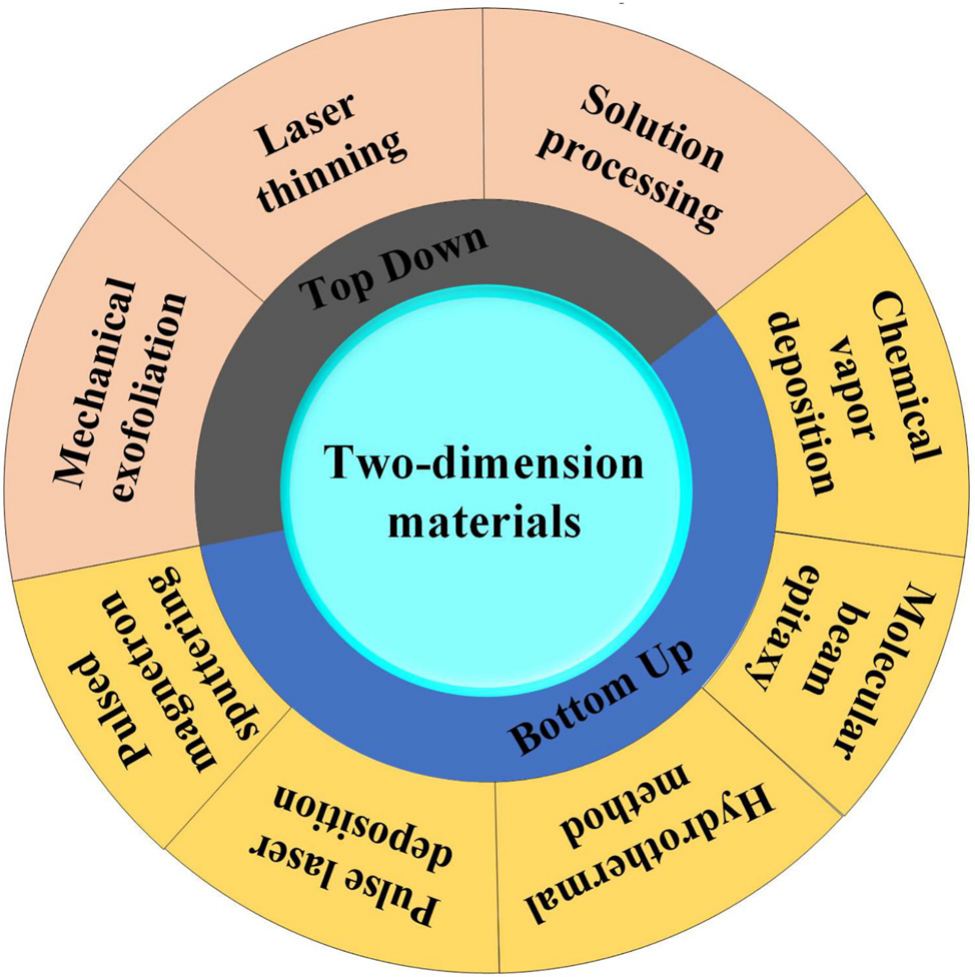
Schematic of the detailed fabrication methods of 2D materials.

The fabrication methods of layered two-dimensional materials can be roughly divided into two types: Top-down exfoliation and Bottom-up growth. Figure 3 summarizes the detailed fabrication methods. This section will mainly introduce three commonly used methods.

#### 3.2.1 Mechanical exfoliation

Since graphene was exfoliated from graphite by mechanical exfoliation in 2004 [1], this method has been widely used due to its low cost. In addition to the fabrication of graphene, high-quality single-crystal flakes of two-dimensional materials such as black phosphorus have also been successfully fabricated by this method [98–102]. The mechanical exfoliation method usually uses transparent tape to repeatedly stick the surface of the material to obtain a small amount of two-dimensional material. This method is simple to implement, and the obtained single-layer or few-layer materials has high integrity and few defects, so it is suitable for basic scientific research. However, this method is less efficient when a certain amount of 2D material is required due to less 2D materials fabricated each time [102].

#### 3.2.2 Liquid phase epitaxy (LPE)

Liquid phase epitaxy is to mix powdered materials with solvents, and generate bubbles through long-term and high-intensity ultrasound to pulverize the materials into single-layer or several-layer nanosheets. After centrifugation, the small-sized nanomaterials are suspended in the solvent, and the large-sized materials sink to the bottom of the solvent to achieve the effect of separation [103, 104]. Finally, the nanomaterials on the upper layer of the solvent are collected with a pipette, and this method does not require post-processing. In 2015, the liquid phase epitaxy method was used to fabricate BP for first time [105], and excellent nanosheet structure and Z-scan curve were obtained. This method has high yield and low cost [106], but it is difficult to control the number of layers and size of 2D materials by this method. Although single-layer nanosheets can be generated, the concentration is typically lower compared to several-layer nanosheets [107].

#### 3.2.3 Chemical vapor deposition (CVD)

**Figure 4:**
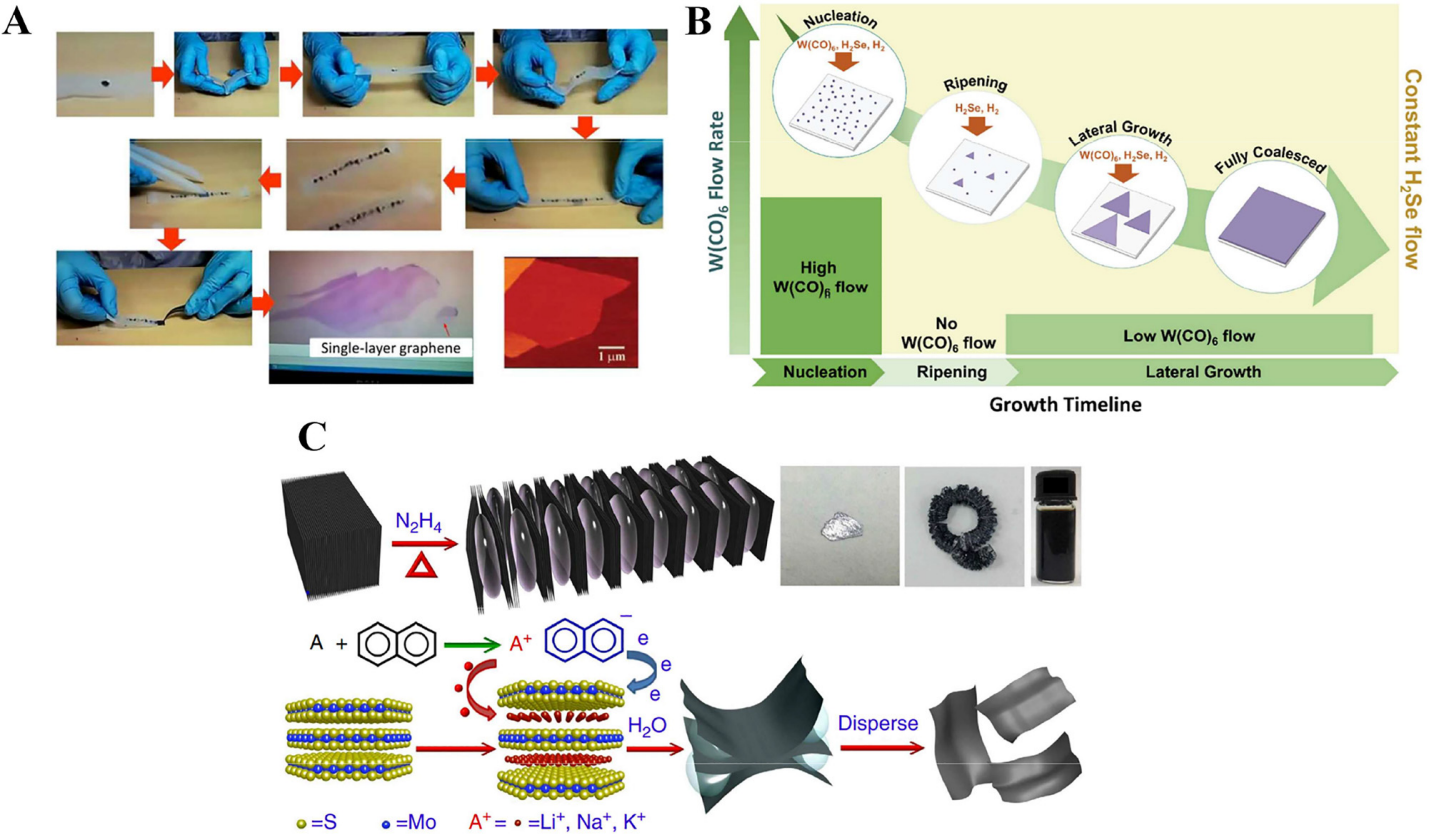
Typical fabrication methods of 2D materials. (A) The illustrative procedure of Scotch-tape based micromechanical cleavage of HOPG. Reproduced with permission from Ref. [99]. Copyright 2015, Royal Society of Chemistry. (B) Schematic diagram showing the multistep process of single-layered sheets synthesis of WSe_2_ using chemical vapor deposition. Reproduced with permission from Ref. [112]. Copyright 2018, American Chemical Society. (C) Schematic exhibiting the multistep alkali-metal based intercalation liquid phase exfoliation method to obtain single-layer sheets in water. Reproduced with permission from Ref. [113]. Copyright 2014, Springer Nature.

Compare with the above two methods, chemical vapor deposition is an important method for fabricating two-dimensional materials. In this method, gaseous and powdered reactants are placed in a tube furnace, where specific chemical reactions occur at suitable temperatures and produce 2D materials on a substrate downstream of the tube furnace [108]. The chemical vapor deposition method can control the size and number of layers of the two-dimensional materials produced by modifying the parameters. The fabricated materials have the advantages of large lateral size and few defects, which facilitates the study of their intrinsic properties. Moreover, the high yield and controllability of this method make it the main method for large-scale commercial production [109]. This method has been successfully used for the fabrication of MXenes, transition metal tungsten, tantalum, and other materials [110, 111]. The experimental schematic diagram of the above three methods is shown in Figure 4.

### 3.3 2D material-integrated microfiber photonic device fabrication

At present, there are two commonly used methods to decorate microfiber with two-dimensional materials. One is to use the optical gradient force induced by the strong evanescent field of the microfiber to deposit two-dimensional materials on the waist of the microfiber. The second is to transfer the prepared 2D material film/sheet to the waist of the microfiber for covering/wrapping, or attach the microfiber to the 2D material film/sheet.

#### 3.3.1 Deposition method

**Figure 5:**
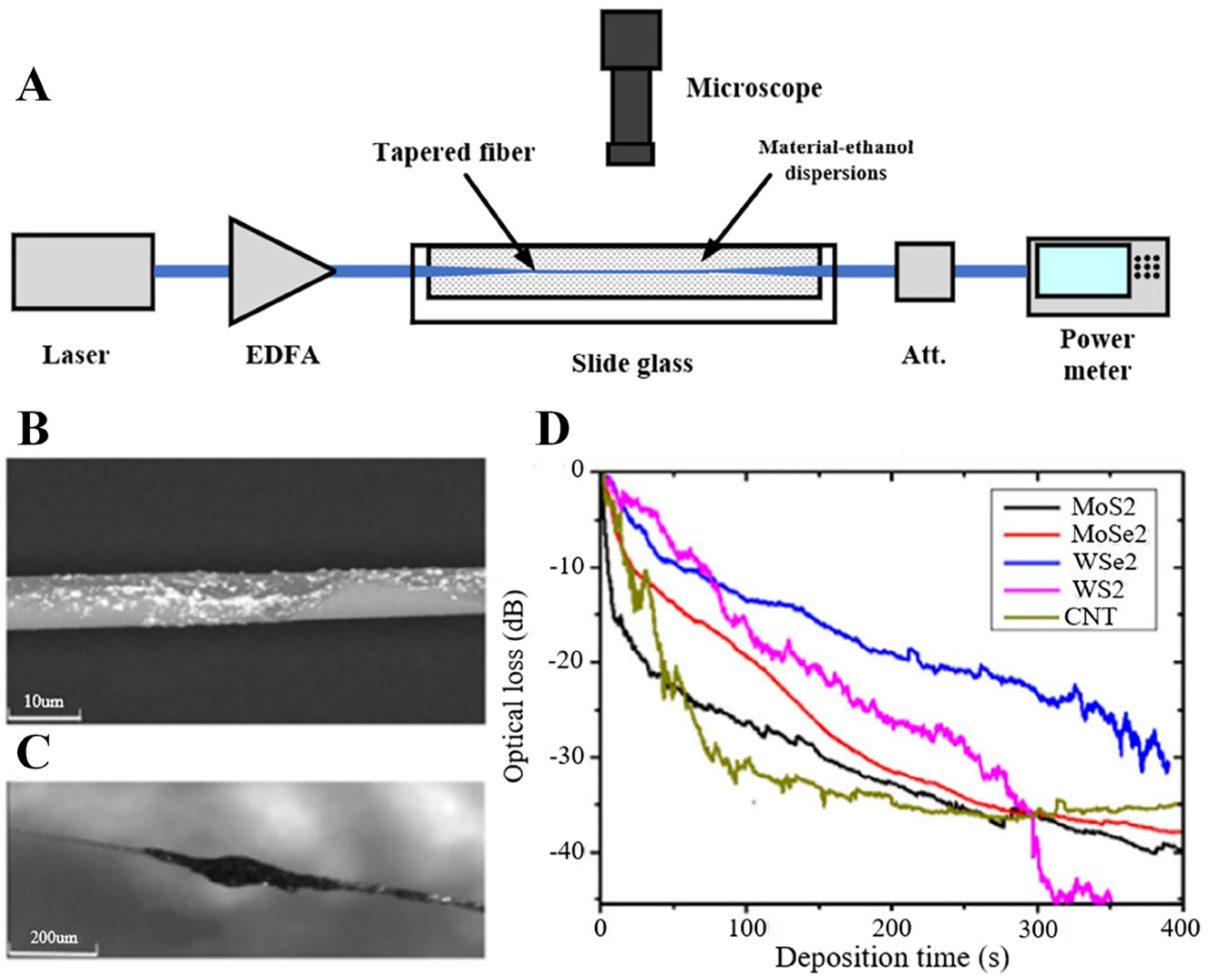
2D material-decorated microfiber photonic device fabrication using ECD method. (A) Experimental setup of ECD method. (B) Microscopic image of a MoSe_2_ deposited tapered fiber. (C) SEM image of a MoSe_2_ deposited tapered fiber. (D) Deposition curves of CNTs, MoS_2_, MoSe_2_, WS_2_ and WSe_2_. Reproduced with permission from Ref. [114]. Copyright 2018, Elsevier.

An experimental setup using ethanol to catalyze optical deposition is shown in Figure 5A, this deposition method is similar to traditional optically driven deposition, but the minimum threshold power required is as low as 0 dBm [114]. The material to be deposited is prepared by liquid phase epitaxy. First, the tapered fiber is immersed in the material, when light is injected into the fiber, the light source is amplified by an erbium-doped fiber amplifier to obtain a stronger evanescent field. The light field will attract nanomaterials to attach to the tapered fiber. The output power of the tapered fiber is monitored by an optical power meter. This method uses ethanol as a solution of the dispersant, and the high volatility of ethanol significantly promotes the Brownian motion of the material nanosheets in the dispersion, making the material easier to deposit on the tapered fibers. The slide has a deep groove in the middle, allowing the tapered fibers to be fully immersed in the dispersant during the deposition process. The deposition quality can be optimized by controlling the optical power and deposition time, and the remaining 2D material solution is taken out by a pipette, and finally the prepared 2D material-integrated microfiber are evaporated at room temperature. The strong interaction between 2D materials and evanescent fields may cause optical damage to 2D materials and microfiber, and microfiber with larger diameters are beneficial to reduce the interaction effect and insertion loss. The traditional optical deposition method is a more common method currently, which is simple to operate, but usually requires an optical amplifier to amplify optical power to drive the deposition process, and it is difficult to achieve uniform material thickness during the photonic period fabricated by this method because it is difficult to control the deposition process, the concentration of the material, and the deposition length are difficult to unify [115].

#### 3.3.2 Covering/attaching/wrapping

**Figure 6:**
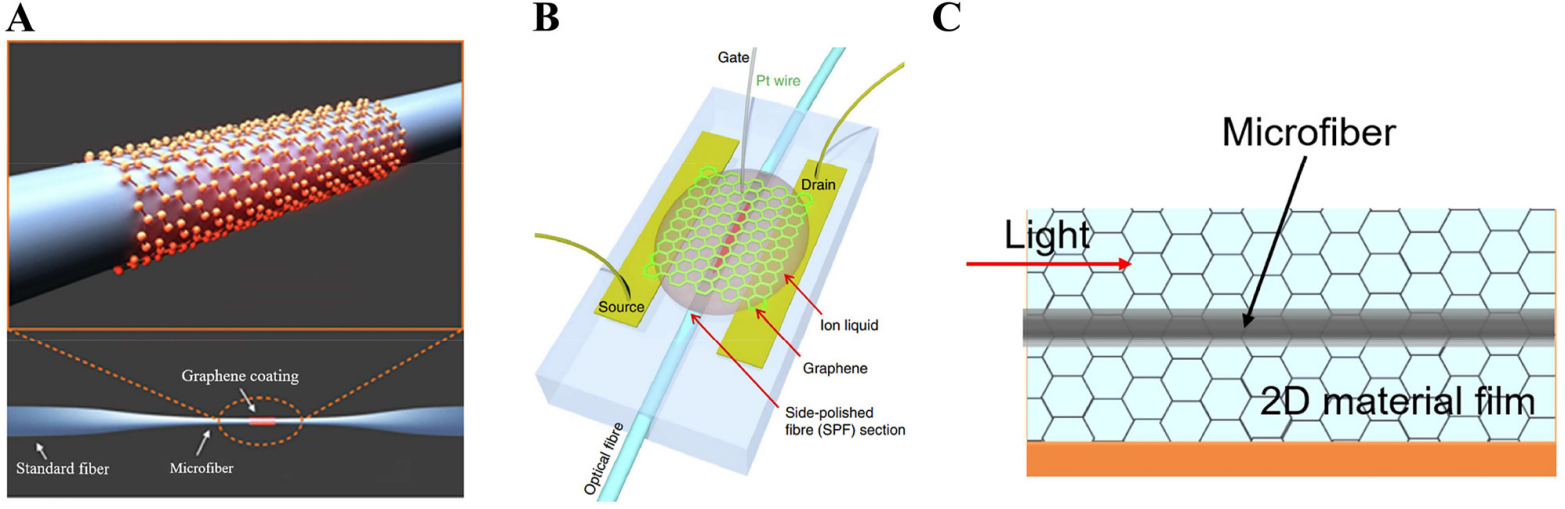
2D material-decorated microfiber photonic device fabrication using the wrapping/covering/attaching method. (A) Graphene-wrapped microfiber. Reproduced with permission from Ref. [116]. Copyright 2014, American Chemical Society. (B) Graphene-covered D-shaped fiber. Reproduced with permission from Ref. [117]. Copyright 2015, Springer Nature. (C) Microfiber-attached 2D materials.

In addition to fabricating the two-dimensional materials-integrated microfiber composite waveguide by optical deposition, the composite waveguide can also be fabricated by covering, wrapping, or attaching methods. The covering method is shown in Figure 6B [117], and it is to prepare two-dimensional materials into thin films and transfer them to the surface of microfiber for covering; the attaching method is shown in Figure 6C, which is to attach the microfiber to the two-dimensional material film; The wrapping method is more complicated. Figure 6A shows the structure of graphene-wrapped microfiber [116], and the fabrication process is as follows. First, the graphene sheet is pasted onto the microfiber, removed the tape, and the graphene sheet is cut to a width of 10 μm by a nanosecond pulsed laser beam. Finally, when the microfiber is stripped from the glass slide, the graphene flakes would spontaneously wrap around the microfiber to form a composite waveguide. When such preparation methods are used, it is usually necessary to prepare high quality single-layer or multi-layer 2D material films, and the method of preparing the films is very complicated. Compared to optical deposition method, use these methods can obtain photonic devices with uniform and high-quality surfaces.

## 4 Application of two-dimensional material-integrated microfiber photonic devices in all-optical signal processing

**Figure 7:**
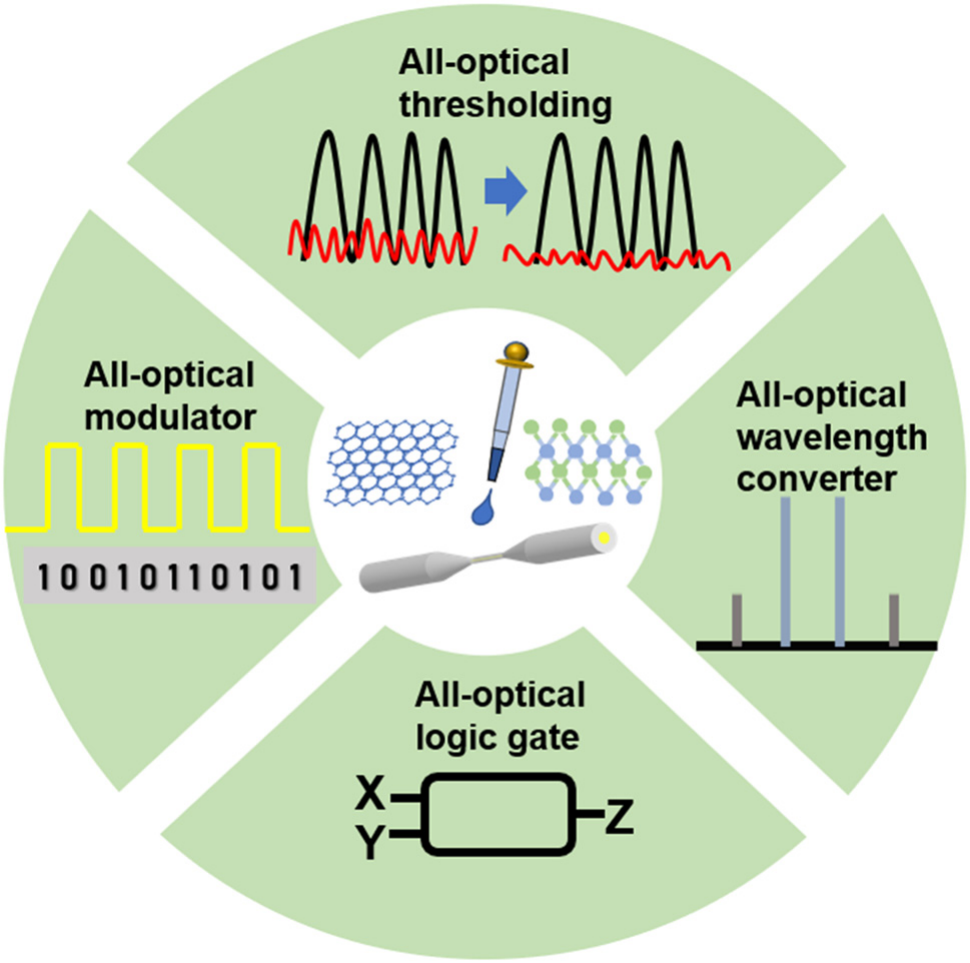
All-optical signal processing technology based on composite waveguide.

All-optical signal processing is an indispensable key technology in the realization of high-speed optical communication networks, mainly including all-optical modulation, wavelength converter, all-optical regeneration, and all-optical logic gates, etc. Provide effective solutions for all-optical routing, all-optical switching, and conflict resolution in future optical communication networks. In the following, the application of 2D material-integrated microfiber photonic devices in all-optical signal processing will be briefly described (as shown in Figure 7).

### 4.1 All-optical modulator

In the all-optical signal processing technology, all-optical modulation technology, which modulates the propagation state of the signal light is one of the most fundamental and critical technologies [118]. The most commonly used modulators in current communication systems are electro-optic modulators and thermo-optic modulators. However, with the demand for modulation speed, traditional optical modulators have reached a bottleneck and new modulation methods are being sought. All-optical modulators use one light to control another light, and change the refractive index of the material by injecting external stimuli such as temperature, pressure, light field, or electric field into the material, thereby changing the signal transmission state of the light (intensity, phase, polarization). Compared with traditional bulk materials, 2D materials exhibit excellent nonlinear optical responses, such as extremely broadband optical responses, less scattering losses, and high-speed carrier responses [119, 120], this in turn makes it possible to realize light modulation at nanoscale [121, 122]. All-optical modulators have many fundamental advantages. They have low insertion loss. In addition, compared to the lattice mismatch problems of bulk materials, 2D materials can be easily incorporated into microfibers. The natural compatibility of microfiber-integrated optical modulators with commercial communication systems makes all-optical modulators have great potential, and are considered to be the most promising for practical applications [123–125]. In this section, the current all-optical modulators based on two-dimensional material-integrated microfiber composite waveguides are mainly reviewed, and they are classified according to their modulation principles. All-optical modulators based on the three effects of saturable absorption, Kerr effect, and thermo-optic effect of two-dimensional materials are reviewed.

#### 4.1.1 All-optical modulator based on saturable absorption

The saturable absorption effect is a third-order nonlinear effect, which describes the absorption of light by a material depending on the intensity of the incident light. The effect is derived from the Pauli blocking or energy band filling effect in two-dimensional materials [126]. When the signal laser transmitted in the composite waveguide is weak, it will be absorbed by the two-dimensional materials to produce obvious attenuation, while when switching light is introduced, it will excite the carriers in the 2D material, and the excited carriers will lead to the band filling effect, change the absorption spectrum, shift the absorption threshold of the 2D materials to a higher frequency, and reduce the attenuation of the signal light, and the switching light will lead to the modulation of the signal light [116]. Many two-dimensional materials exhibit excellent saturable absorption properties, such as graphene, which has a strong saturable absorption with ultra-fast response [22].

**Figure 8:**
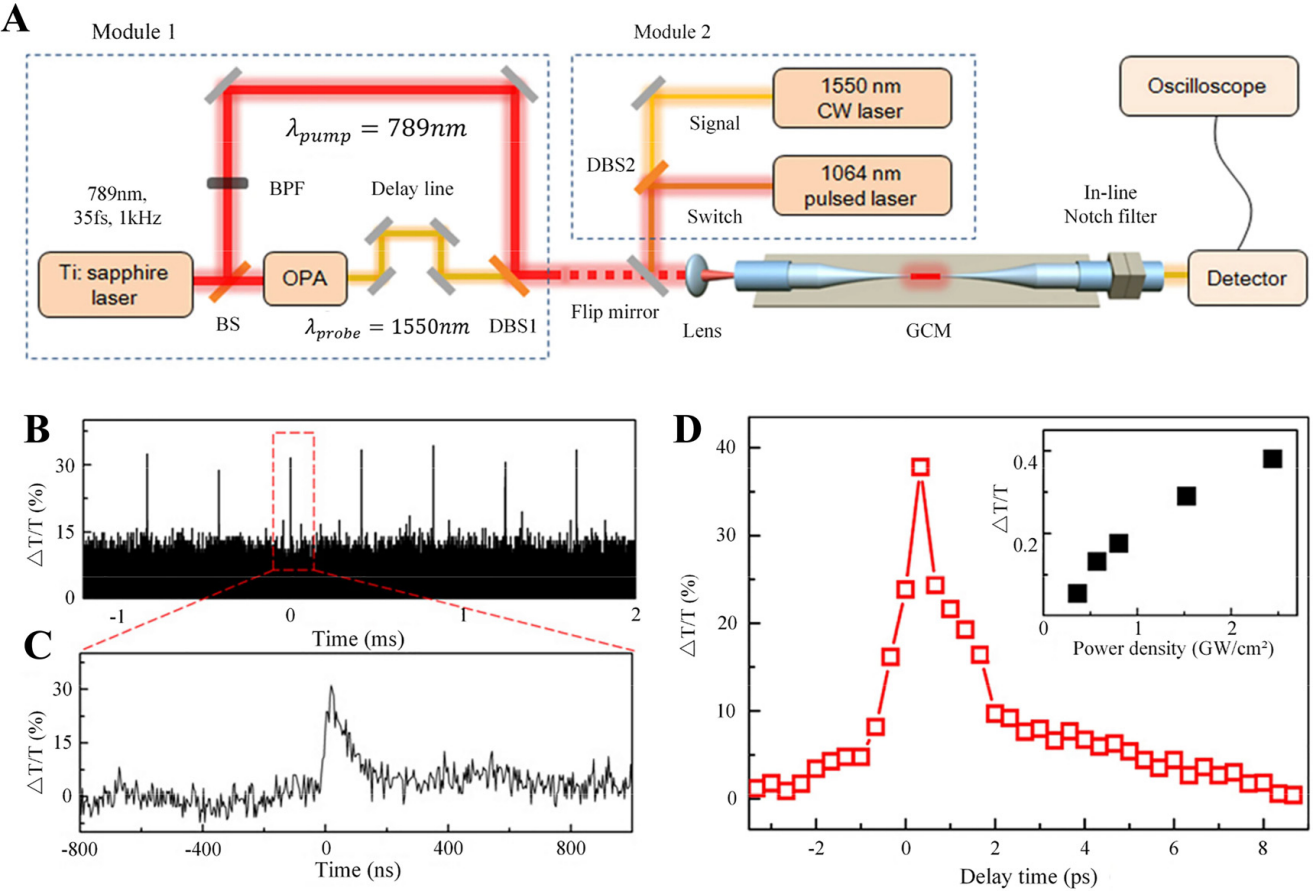
All-optical, all-fiber graphene modulator based on GCM. (A) Schematic illustration of all-optical modulation setups. (B) Pulses switched out from a 1550 nm CW beam in a GCM by a 5 ns 1064 nm pump pulse train. The induced differential transmittance is ∼30 %. (C) Time profile of a switched-out pulse. (D) Differential transmittance of the probe light through a 1.4 μm GCM with 20 μm long graphene cladding as a function of the pump-probe time delay with a pump power of 200 nW, showing a response time of ∼2.2 ps. The inset shows the dependence of the modulation depth on pump intensity. Reproduced with permission from Ref. [116]. Copyright 2014, American Chemical Society.

In 2014, an all-fiber graphene modulator was demonstrated [116], the structure is shown in Figure 8A. It is made by wrapping a thin layer of graphene around a microfiber (about 1 μm in diameter at the waist) drawn from a standard single-mode fiber. As the light field is confined in the microfiber, when switching light is introduced, it excites the carriers in the graphene, which shifts the graphene’s absorption threshold to higher frequencies with the Pauli blocking of interband transitions, resulting in much lower attenuation of signal light, realize efficient modulation of signal light. The response time of the modulator is about 2.2 ps, corresponding to a calculated bandwidth of about 200 Ghz for Gaussian pulses. It is limited only by the intrinsic graphene response time. In addition, the modulation depth of the modulator can reach 38 %. One of the main ways to increase the modulation depth is to increase the interaction distance of the light with the 2D materials. High-length interaction distance achieved by winding microfibers on glass rods with graphene deposited on the surface, the modulation depth is as high as 7.5 dB [127]. However, as the interaction distance between graphene and microfiber increases, the insertion loss of the device will also increase. By optimizing the structure and the composition of the two-dimensional materials, the performance of this device in all-optical modulator can be expected.

However, the low modulation depth and overall transmission resulting from the strong linear absorption and modulation scheme in graphene limit the applications of such optical modulators. Researchers are gradually focusing on other emerging two-dimensional materials.

**Figure 9:**
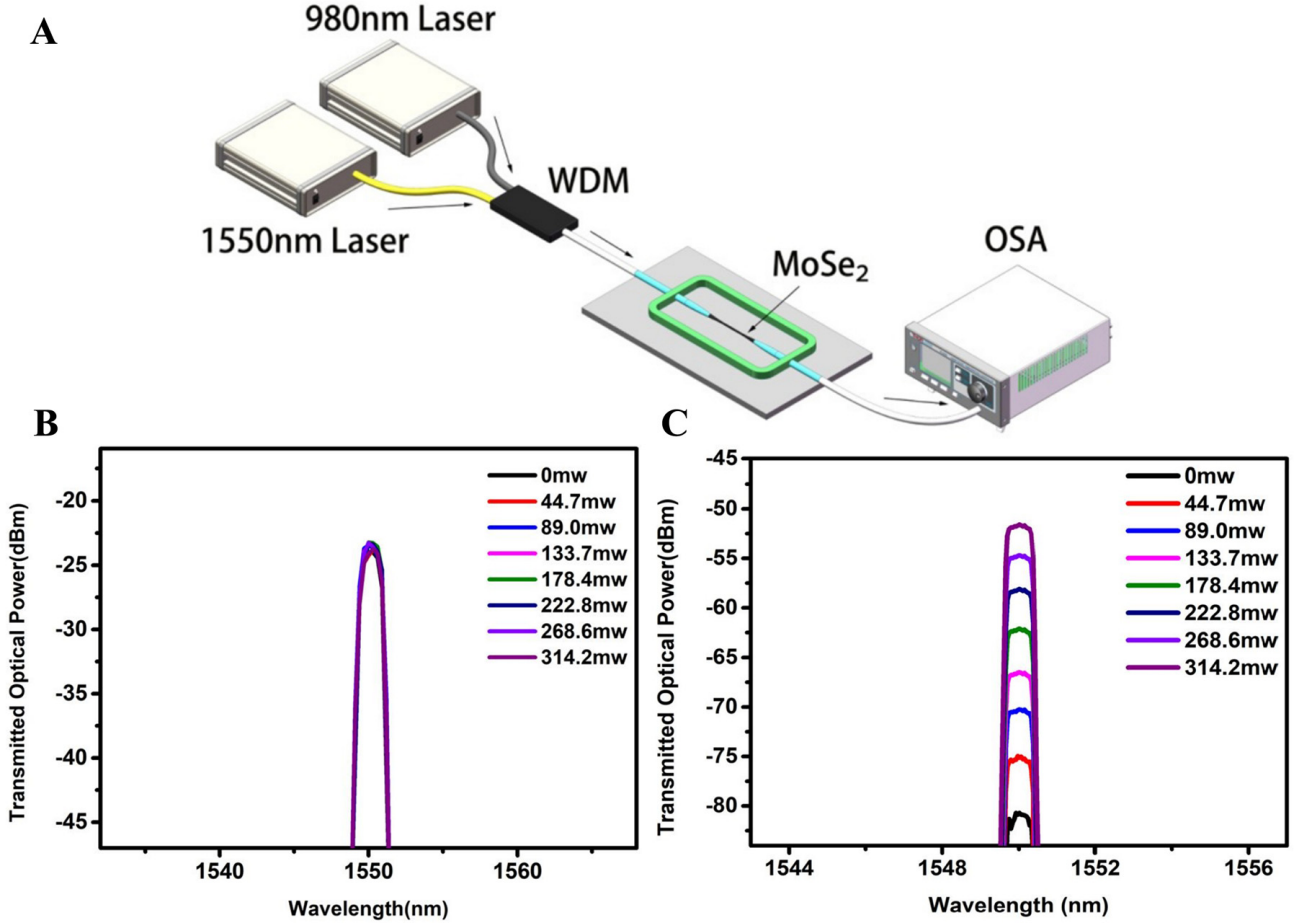
All-optical modulator based on microfiber coated with MoSe_2_. (A) Schematic of 980 nm in-fiber pumped experimental setup for the MF coated with MoSe_2_. (B) The optical transmitted power of the MF without MoSe_2_ for different pump power. (C) The optical transmitted power of the MF with MoSe_2_ for different pump power. Reproduced with permission from Ref. [128]. Copyright 2019, The Optical Society.

In 2017, Zhang et al. demonstrated a light-control-light experiment based on MoSe_2_-coated-microfiber, using 980 nm pump light to modulate the signal light by pumping inside the fiber and pumping outside the fiber respectively [128]. Figure 9A is the structure diagram of 980 nm in-fiber pumped experimental setup. A 1550 nm Distributed Feedback Laser (DFB) laser was used as a signal source coupled with pump light, and the transmission power through the microfiber was measured by a spectrometer. Using the bare microfiber and the microfiber deposited with MoSe_2_ as a comparison, the output power diagrams passing through the microfiber are shown in Figure 9B and C. When the pump power increases from 0 mw to 314.2 mw, the transmitted power in the bare microfiber is almost unchanged; however, the emission power in the microfiber deposited with MoSe_2_ changes by about 30 dB, and the power of the transmitted light increases with the increase of the 980 nm pump power. The sensitivities to 980 nm pump light in-fiber pump and out-fiber pump are 0.092 dB/mW and 0.851 dB/mW respectively, the corresponding rise time and fall time of the transient are 0.4 s and 0.6 s, respectively. The principle of light-control-light is that the guided light in the microfiber is absorbed by MoSe_2_, and the concentration of excited electron holes in MoSe_2_ increased with the increase of the pump power, resulting in a decrease in the actual part of the dynamic conductivity [129], reducing the absorption of the guided light by MoSe_2_ nanosheets, thereby increasing the transmission power of microfiber [130].

#### 4.1.2 All-optical modulator based on Kerr effect

The optical Kerr effect describes the change of the refractive index in a non-linear medium, where the change of the refractive index is proportional to the intensity of the incident light. Ultrafast phase modulation requires phase modulation using the Kerr effect. The optical Kerr effect leads to many phenomena, such as four-wave mixing, self-phase modulation, etc. 2D materials have a good Kerr nonlinearity, and the nonlinear refractive index of two-dimensional materials is several orders of magnitude larger than that of the former compared to the bulk materials.

**Figure 10:**
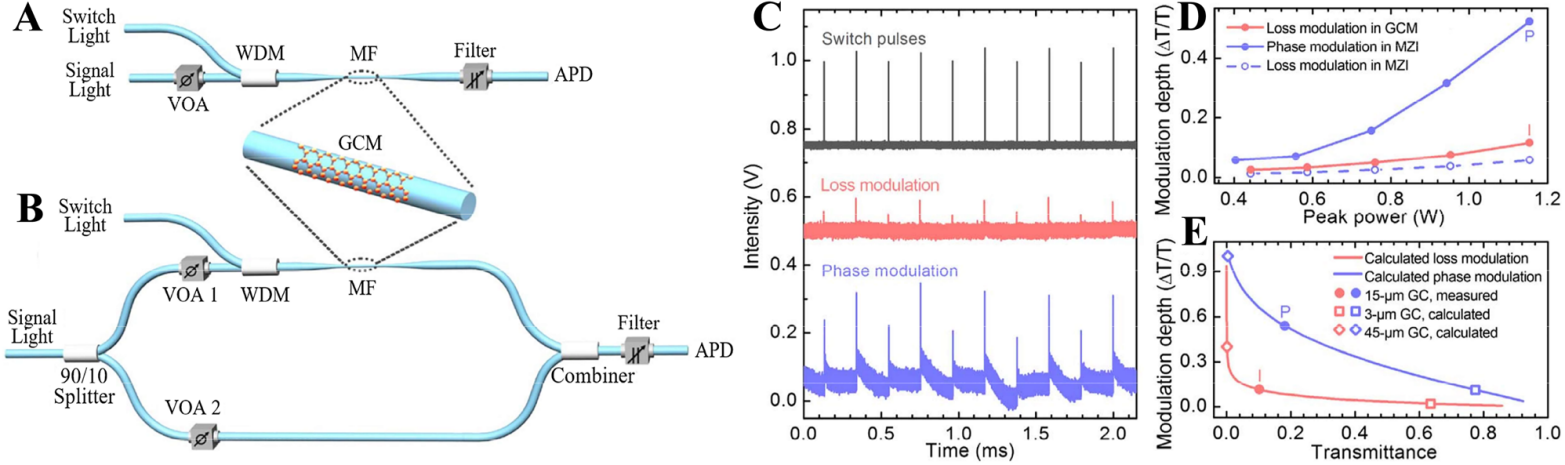
GCM-based all-optical modulators. (A) Schematics of GCM-based all-optical modulators based on optically induced loss modulation in a GCM. (B) Schematics of GCM-based all-optical modulators based on optically induced phase modulation in a GCM in one arm of an all-fiber MZI. (C) Top panel, pairs of switching pulses; middle panel, pulse-modulated signal from a GCM-contained fiber; bottom panel, pulse-modulated signal from an MZI as a result of phase modulation in the GCM-contained arm. (D) Modulation depth of the output signal as a function of the peak switching power for the GCM-contained fiber modulator (red solid line), for the MZI modulator (blue solid line), and for the MZI modulator with only loss modulation in the GCM-contained arm of the MZI (blue dashed line). (E) Modulation depth versus overall transmittance (OT) for the GCM-contained fiber modulator (red line) and the MZI modulator (blue line) with different graphene cladding lengths as indicated. Reproduced with permission from Ref. [78]. Copyright 2016, The Optical Society.

In 2016, Yu et al. proposed an all-optical graphene modulator based on optical Kerr phase shift (as shown in Figure 10) [78], the total transmittance of MZI-based modulators is significantly higher than that of the graphene loss modulator, and the modulation depth reaches 4.6 times of the latter [116]. In the experiment, 1550 nm Continuous wave (CW) laser is used as the signal light, and 1064 nm pulsed laser is used as the switching light. By controlling the switching light, the nonlinear effect in graphene modulated the intensity and phase of the signal light at the same time, the loss modulation is caused by the saturable absorption effects. In order to minimize the influence of the saturable absorption effect, the signal power injected into the two arms of the MZI was changed to 90:10. Figure 10C shows the switch, loss modulated, and phase modulated pulses. As shown in Figure 10D, as the pump power increases, the modulation depth of the phase modulation also gradually increases. In this system, the phase modulation plays a leading role, and the output of the system is the signal light modulated by the optical Kerr effect.

**Figure 11:**
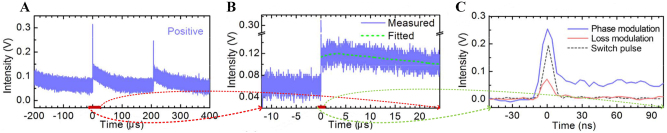
Temporal profile of the modulated signal. (A) and (B) Temporal profiles of pulse-modulated signals from a MZI modulator in different time scales. (C) Temporal profiles of pulse-modulated signals from a MZI modulator and a GCM modulator on the nanosecond time scale in comparison with the temporal profile of the switching pulse. Reproduced with permission from Ref. [78]. Copyright 2016, The Optical Society.

Enlarge the pulse waveform of phase modulation in Figure 10C, as shown in Figure 11, the pulse modulation signal peak consists of a nanosecond pulse and a tail with a decay time of about 100 µs, and the nanosecond pulse comes from the refractive index change caused by carrier excitation in graphene, and the tail comes from the refractive index change caused by the thermal-optical effect in graphene. The accumulation of thermal effect may limit the performance of all-optical modulators based on two-dimensional materials. The phase modulator based on the Kerr effect has a faster response time, and the modulation depth can be further improved by increasing the length of the 2D materials cladding.

#### 4.1.3 Thermo-optic phase shifter ***s*** and thermo-optic switches

All-optical phase shifters and thermo-optic switches both play important roles in applications such as all-optical signal processing, sensing, and communication. The thermo-optic effect is that the refractive index of a material changes with temperature. The change in refractive index causes a phase shift in the transmitted light, so it can be applied to modulation. The thermal conductivity of two-dimensional materials ensures the response time of the phase shifter. Two-dimensional materials such as graphene are widely used in thermo-optic phase shifters because of their good thermo-optic conversion efficiency and thermal conductivity, which can be used as efficient thermal generators and conductors.

Thermo-optic phase shifters are mainly realized by MZI, Michelson interferometer (MI) and Micro knot resonator (MKR) structures. Combining two-dimensional materials with microfiber has been widely used in thermo-optic phase shifters and thermo-optic switches [105, 131], [132], [133], [134]. In the following, we will introduce the thermo-optic modulators and thermo-optic switches that apply the above three structures.

In 2015 Gan et al. designed and demonstrated an all-optical phase shifter based on graphene thermo-optic effect [131], and the graphene is heated by the pump light of 980 nm and 1540 nm, and the refractive index of the fiber is changed through the thermo-optic effect, thereby changing the interference phase shift of the MZI. As for the phase shifter, the characteristic of graphene’s uniform absorption in a wide spectrum will lead to a large loss at the signal light, which is what researchers do not want to see. However, WS_2_ has good absorption at 980 nm and weak absorption at 1550 nm, which makes it one of the candidate materials for phase shifters [132].

**Figure 12:**
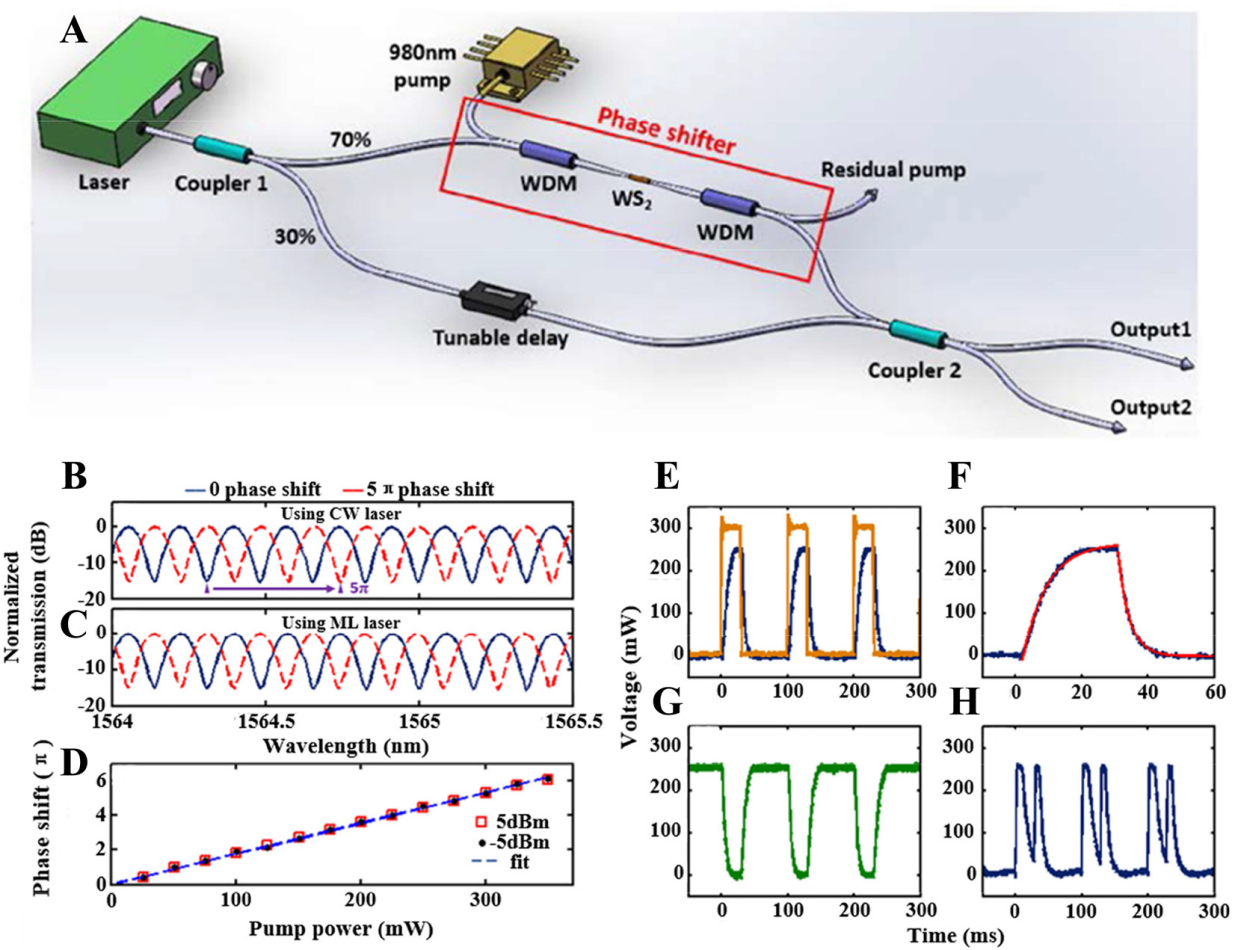
All-optical phase shifter and switch based on WS_2_ deposited tapered fiber. (A) Experimental setup. (B–D) Transmission spectra of the MZI with 0 (blue) and 5*π* phase shift (red) using (B) a continuous wave laser source and (C) a mode-locked laser source. (D) Phase shift with respect to the injected pump power with two input power levels. (E) Pulsed pump light (yellow) and switch output at “output 1” port (blue). (F) A zoomed view of a single off-on-off transition of the switch (blue) and exponential fit (red). (G) Complementary output at “output 2” port. (H) Output breaking when the MZI is overdriven. Reproduced with permission from Ref. [132]. Copyright 2017, The Optical Society.

In 2017, Wu et al. designed an all-optical phase shifter by depositing WS_2_ on microfiber, which is a typical structure based on MZI all-optical modulator, as shown in Figure 12A [132]. The system consists of multiple couplers, wavelength division multiplexers and an adjustable delay line to form the two arms of the MZI. The delay line is used to compensate the optical path difference and optical power in the two arms. WS_2_ absorbs the injected 980 nm pump to generate heat, which changes the refractive index of WS_2_ and the tapered fiber, and then changes the phase of the signal light transmitted in the upper arm to achieve the purpose of all-optical modulation. Finally, the MZI structure makes the two signals of the upper and lower arms interfere and output the interference spectrum. The maximum phase shift of 6.1*π* is achieved near 1550 nm, and an all-optical switch with an extinction ratio of 15 dB and a rise time of 7.3 ms is obtained. The rise time constant and fall time constant of all-optical switches depend on different parameters, the rise time mainly depends on the ability of 2D materials to absorb control light to change temperature, which is affected by environmental factor, and the fall time depends on the heat dissipation speed of the heated microfiber.

The transmission curves of the two arms of the MZI are cosine functions:

$$\left(1+\cos\Delta \varphi \right)/2,\;\left(1-\cos\Delta \varphi \right)/2$$

It can be seen from the function that the switching can be realized with the change of the phase difference. The situation in Figure 12H is due to the fact that when the instantaneous power of the pump light continues to increase, the phase difference exceeds 2*π*, at this point the MZI is reverse biased, so the input square wave pump pulse is broken into two output pulses with the trough in the middle.

**Figure 13:**
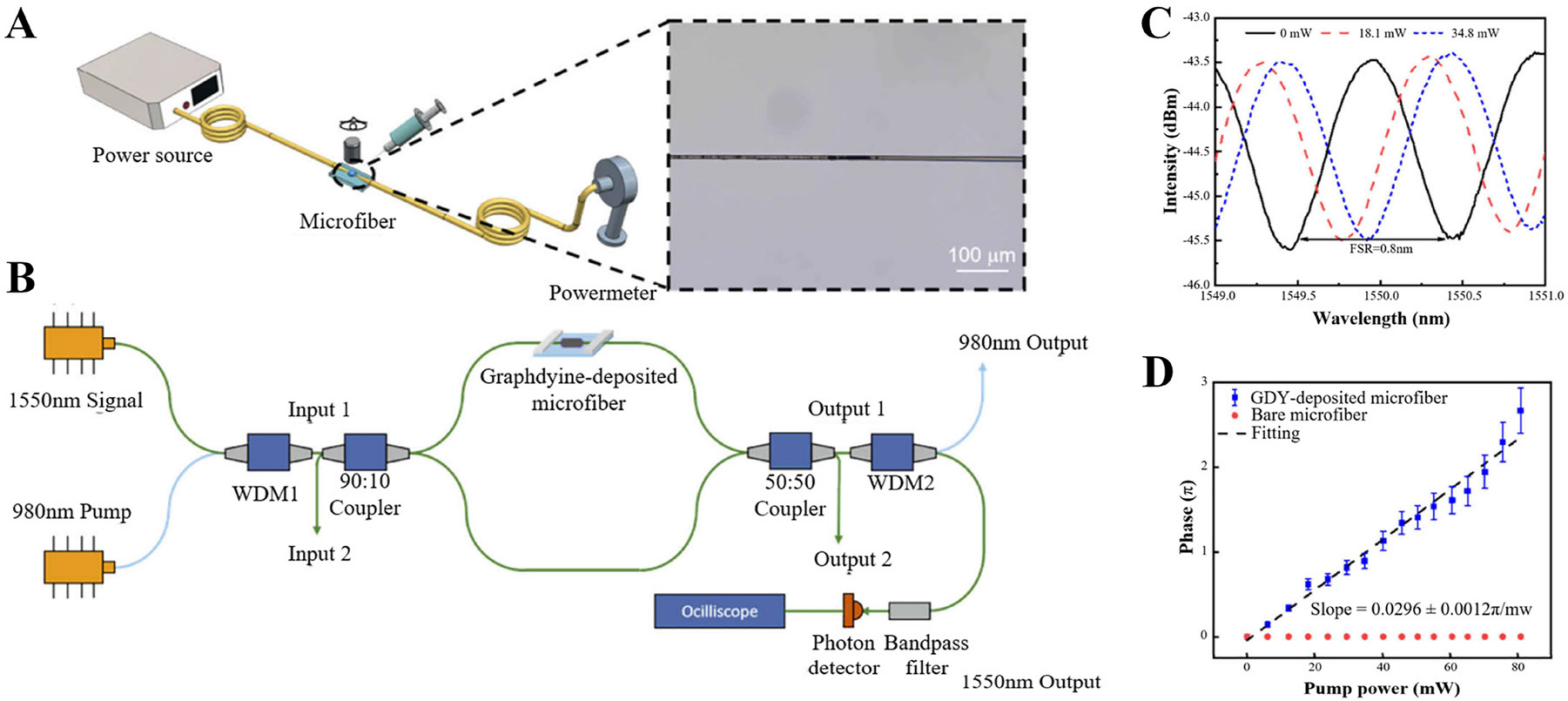
All-fiber phase shifter based on a Graphdiyne-deposited microfiber. (A) Schematic diagram of optical deposition method and optical microscopic image of the GDY-deposited microfiber. (B) Experimental setup of the all-optical modulator. (C) Measured interference spectrum with increasing pump power. (D) Phase shift versus the pump power. Reproduced with permission from Ref. [136]. Copyright 2021, The Optical Society.

Compared with graphene, Graphdiyne (GDY) has higher thermo-optic conversion efficiency and thermal conductivity than graphene [135], making it an excellent thermo-optic material, gradually attracting the attention of researchers. In 2021, Zhang et al. designed and demonstrated a Graphdiyne-deposited microfiber all-optical phase modulator (as shown in Figure 13B) [136]. When the repetition frequency of the pump light is 25 Hz, the corresponding phase conversion efficiency is 0.0296*π* mW^−1^, the rise time constant is 5.48 ms, and the free spectral range (FSR) is 0.8 nm. It is proved by calculation that the normalized phase conversion efficiency of GDY is higher than that of all-optical modulators based on graphene, MXenes and WS_2_, demonstrating the potential of GDY in all-optical modulation devices in the telecom band. GDY also has great potential for ultrafast laser generation, such as the use of GDY deposited tapered fiber as a saturable absorber to generate conventional soliton and noise-like pulses [137].

**Figure 14:**
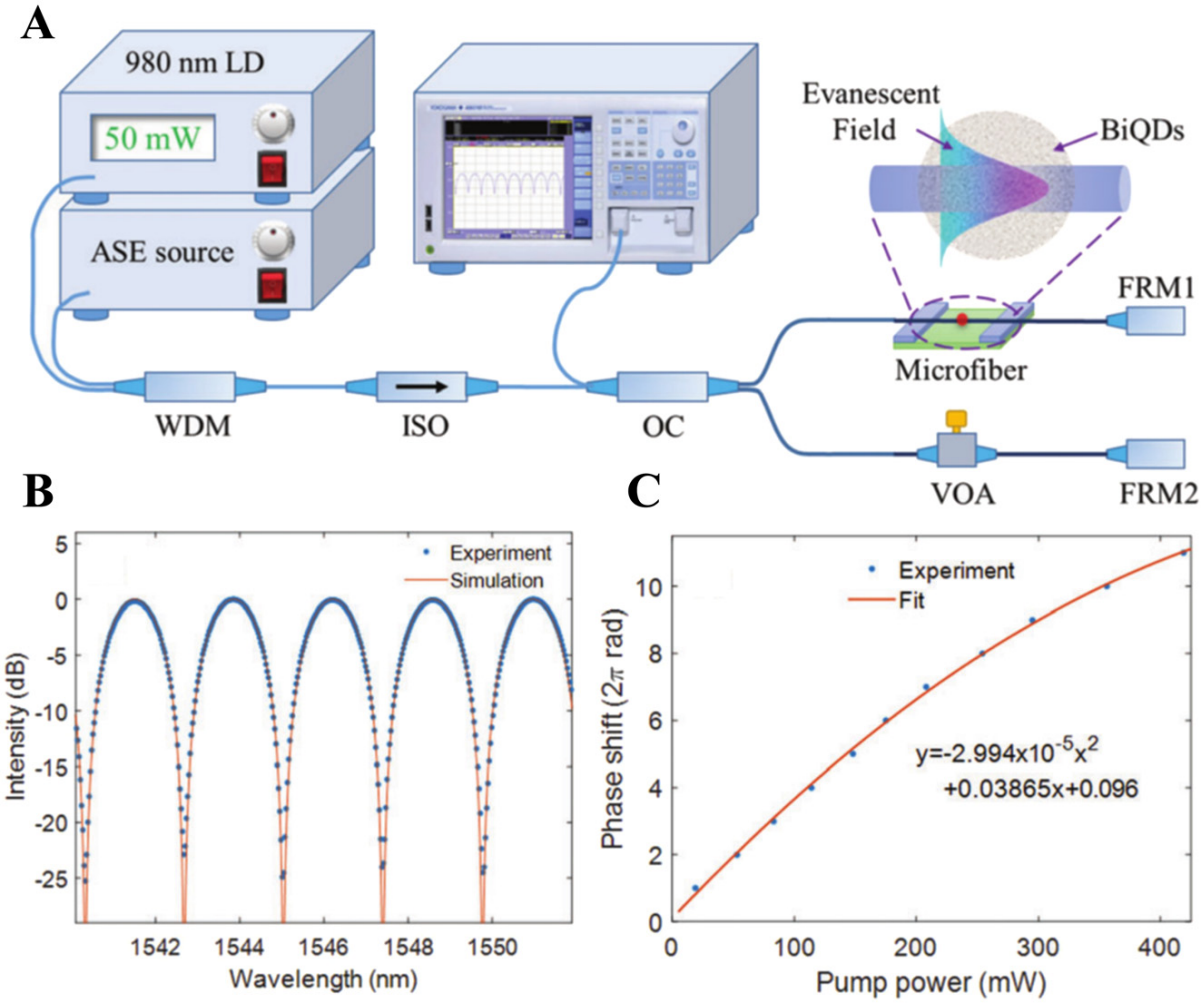
All-optical phase modulator based on a Michelson interferometer. (A) Schematic diagram of the all-optical phase modulator based on BiQDs-deposited-microfiber;(B) The measured interferometric spectrum (blue dots) and theoretical simulation curve (orange line;) (C) Phase shift at different pump powers. Reproduced with permission from Ref. [138]. Copyright 2019, Royal Society of Chemistry.

The all-optical modulators in the above experiments are based on the MZI structure, and one of the main problems of the MZI modulator is that it is very sensitive to temperature changes and stress disturbances, resulting in poor environmental stability and serious polarization fluctuations. Compared with the all-optical switch of the MZI structure, the switch based on the MI structure is relatively stable. Because of the existence of Faraday Rotator Mirror (FRM), the signal light will pass through the all-optical device twice, while improving the effect of the thermo-optic effect, it will also cause a greater phase shift of the signal light, shortening the response time of the system, and eliminates the polarization problem. In 2019, Wang et al. proposed a new all-optical modulator, which deposited bismuth quantum dots prepared by the LPE method onto microfiber to obtain a bismuth-microfiber composite structure (as shown in Figure 14A) [138]. The MI structure controls the thermo-optic effect of light and bismuth quantum dots to change the phase of the signal light on the upper arm. The modulated and unmodulated signals interfere in the coupler, and finally output. The free spectral range is 2.38 nm. The rise/fall time constants of the output signal are 1.56 ms and 1.53 ms, respectively. The resulting conversion efficiency is reduced from 0.076*π* mw^−1^ to 0.053*π* mw^−1^, resulting in a maximum modulation depth of 25 dB. Figure 14B shows the measured optimal interferometric spectrum with the interferometric contrast larger than 25dB. Figure 14C shows the measured phase shift at different pump powers, which is fitted by a second-order polynomial.

**Figure 15:**
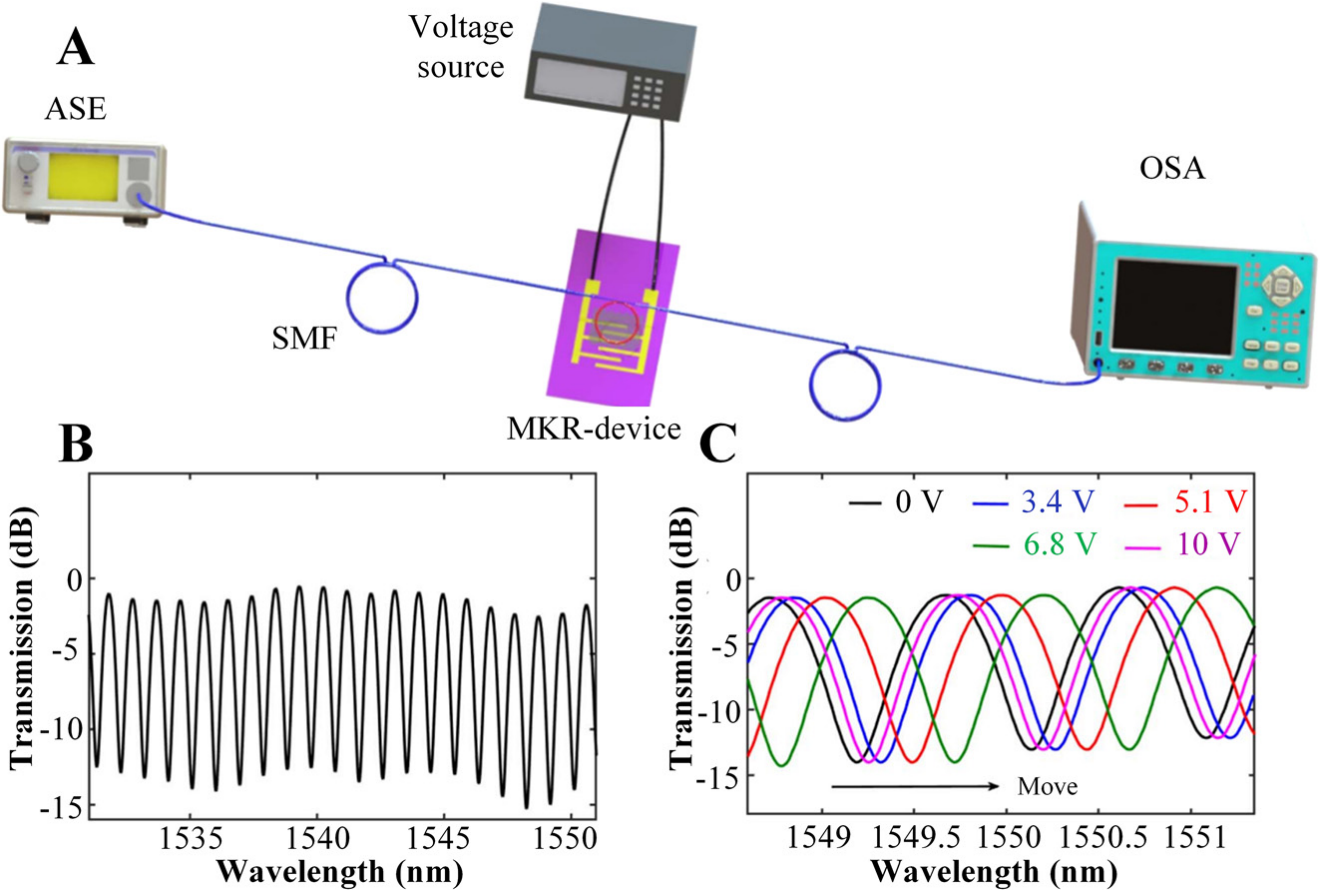
All-optical phase modulator based on MKR structure. (A) Experimental setup of phase modulation system based on the graphene-MKR modulator. (B) Typical transmission spectrum. (C) Transmission spectra under different voltages. Reproduced with permission from Ref. [140]. Copyright 2021, Chinese Laser Press.

However, the MI structure still has limitations and is sensitive to the disturbance of the surrounding environment. Knotting microfibers into rings to form MKR structure, compare with MZI and MI structure, all-optical modulators with MKR structures have the advantages of high stability and small size [139], is a better optical modulation structure. And combined with two-dimensional materials, they are extremely attractive in the application of optoelectronic devices. In 2021, based on the sheet resistance and high thermal conductivity of graphene, Wang et al. proposed a graphene-microfiber knot resonator modulator [140]. The system can realize intensity modulation and phase modulation when narrowband light and broadband light are respectively introduced into the MKR. In the experimental structure (as shown in Figure 15A), a broadband amplified spontaneous emission light source (ASE) was used as the incident light source, and the resonant characteristics of MKR were observed through a spectrometer, by applying a voltage to the graphene to generate a large amount of heat, the thermo-optic effect changes the refractive index of the MKR, thus changes the resonant wavelength of the MKR. The resonant phenomenon of MKR comes from the circulation of part of the incident light in the ring and the interference between the recycled light and the output light. The resonant characteristics of MKR are shown in Figure 15B, and each notch in the spectrum corresponds to a resonant wavelength, the resonant wavelength can be expressed as:

$$\lambda_{\text{res}}=2\pi Rn_{\text{eff}}/m$$

Due to the high thermal conductivity of graphene and the tiny diameter of MKR, a maximum phase shift of 2.1*π* was achieved at the highest available voltage of 10 V. The working principle of MKR modulator as an optical switch is as follows: when the light wavelength is close to the resonant wavelength of the MKR, the output light intensity will be weak; when the light wavelength is far away from the MKR resonant wavelength, the output light intensity will be greater. Therefore, the resonant wavelength of the MKR can be adjusted by applying different voltages to the graphene to control the output light intensity. Compared with other thermo-optic devices, the response time of the modulator based on the MKR structure can reach microsecond level, because its unique resonance structure can improve the heating efficiency. At the same time, it overcomes the shortcomings of the short interaction distance of traditional 2D materials-integrated microfiber. Although thermo-optic modulators have good modulation depth, the modulation rate is on the order of microseconds, which limits the application of high rates. All-optical modulation is one of the most basic technologies in all-optical signal processing, and it also poses a demand for exploring new two-dimensional materials. Table 2 summarizes the thermo-optic phase shifters and thermo-optic switches based on different structures in recent years.

**Table 2:** Comparison of thermo-optic switches with different 2D materials and structures.

Year	2D material	Structure	Phase shift slope (*π* mw^−1^)	RTC	FTC	Ref.
2015	Graphene	MZI	0.091*π* mw^−1^	4 ms	1.4 ms	[131]
2017	WS_2_	MZI	0.0174*π* mw^−1^	7.3 ms	3.5 ms	[132]
2018	BP	MZI	0.029*π* mw^−1^	2.5 ms	2.1 ms	[52]
2019	Graphene	MZI	NA	13.6 ms	22.7 ms	[141]
2021	Graphdiyne	MZI	0.0296*π* mw^−1^	NA	NA	[136]
2019	Antimonene	MI	0.049*π*·mw^−1^	3.2 ms	2.9 ms	[54]
2019	Bismuthene	MI	0.076–0.053*π* mw^−1^	1.56 ms	1.53 ms	[138]
2019	MXene Ti_3_C_2_T_x_	MI	0.043*π* mw^−1^	2.3 ms	2.1 ms	[142]
2020	MXene Ti_2_NT_x_	MI	0.03*π* mw^−1^	NA	NA	[143]
2016	Graphene	MKR	0.057*π* mw^−1^	134 µs	96.5 µs	[144]
2021	Graphene	MKR	NA	41.1 µs	41.2 µs	[140]

### 4.2 All-optical wavelength converter

**Figure 16:**
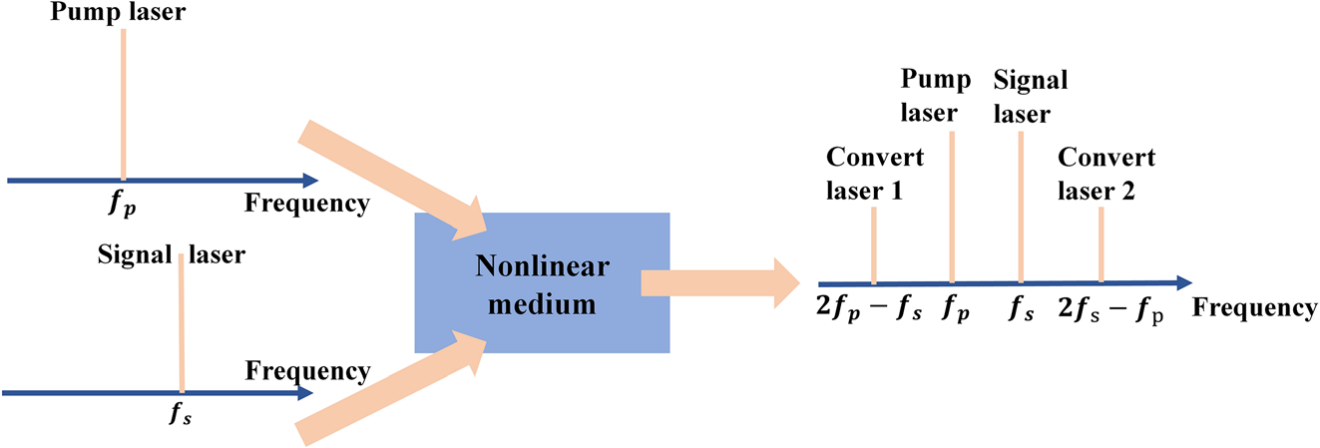
Schematic diagram of all-optical wavelength conversion based on FWM.

All-optical signal processing is a promising and important technology in modern optical communication [50, 51]. The all-optical wavelength conversion technology can directly process optical signals in the optical domain to realize wavelength reuse, which can well avoid the blocking rate of WDM system and effectively utilize bandwidth resources. At the same time, it can give full play to the advantages of high speed and low power consumption of optical signal processing technology. Which plays an important role in solving the wavelength contention problem in the all-optical transmission network, improving the wavelength reuse rate and the flexibility of network configuration. 2D materials, such as graphene, BP, MXenes and TMDs, all have highly nonlinear refractive index, and there is a strong third-order nonlinear optical effect, combined with the high nonlinearity of microfiber, not only will not damage the high-quality waveguide mode, but will induce ultrahigh nonlinear optical response [145–147], enhance the effect of FWM. So they are very suitable for use in wavelength converters [148–151]. Based on the wavelength conversion of four-wave mixing, the converted light can retain the intensity and phase information of the signal light at the same time, and can transparently convert various modulation formats with short response time. At present, most of the all-optical wavelength converters based on two-dimensional materials-integrated microfiber are used the principle of four-wave mixing, the principle is shown in Figure 16, When the signal laser and pump laser are transmitted in the nonlinear medium, if the phase matching condition is satisfied, the FWM effect will be generated, resulting in two converted lasers on both sides of the signal laser and the pump laser. This section will briefly introduce several wavelength conversion experiments based on the principle of four-wave mixing.

In 2014, Wu et al. proved through experiments that the 2 µm microfiber attached to the graphene film can achieve effective four-wave mixing and wavelength conversion [148]. However, with further research by researchers, it was found that some mainstream two-dimensional materials have shortcomings in the application of optoelectronic devices. For example, the limited ability to manipulate light due to the low damage threshold and weak absorption of graphene may limit its application in wavelength converters [152], the instability of black phosphorus in the environment and so on. At present, emerging two-dimensional materials such as MXenes, Antimonene and Borophene are becoming promising nonlinear optical materials due to their good photoelectric properties and long-term stability [86, 153], [154], [155].

**Figure 17:**
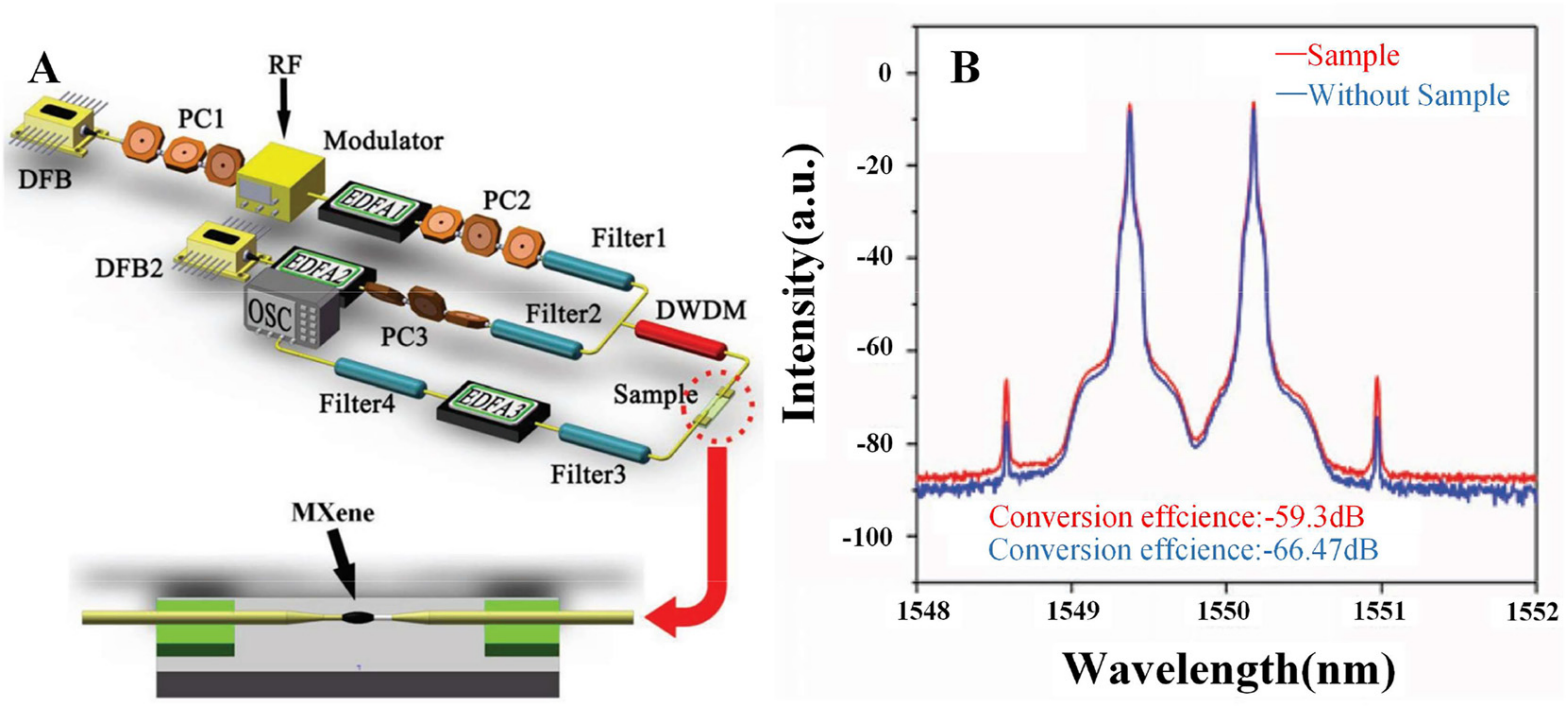
FWM wavelength conversion based on the MXene-decorated microfiber. (A) Experimental setup for the observation of FWM. (B) Effect of the MXene-decorated microfiber on the FWM effect. Reproduced with permission from Ref. [86]. Copyright 2019, John Wiley and Sons.

Compared with graphene, there are fewer studies on the nonlinear optical properties of MXene, mainly focusing on the third-order nonlinear optical effects, and researchers have studied its application in wavelength conversion. In 2019, Song et al. reported an all-optical wavelength converter based on four-wave mixing by depositing MXene Ti_3_C_2_T_x_ on a microfiber [86], the structure is shown in Figure 17A. A DFB laser is used as the signal light, modulated by a radio frequency (RF) modulator, amplified by an erbium-doped fiber amplifier (EDFA), and another DFB laser is used as a pump light to be amplified by another erbium-doped fiber amplifier, a polarization controller was used to control the polarization of the input light in each path to meet the phase matching condition, and the noise amplified by the erbium-doped fiber amplifier is filtered through an adjustable filter. The MXene samples in the experiment were fabricated by liquid acid etching. The length of the sample deposited on the microfiber was about 260 μm, the total length of the microfiber was about 1 cm, and the diameter was about 8 μm. When the input signal laser is 1550.18 nm and the pump light is 1548.58 nm, the two newly generated wavelengths are located at 1546.998 nm and 1551.78 nm respectively.

**Figure 18:**
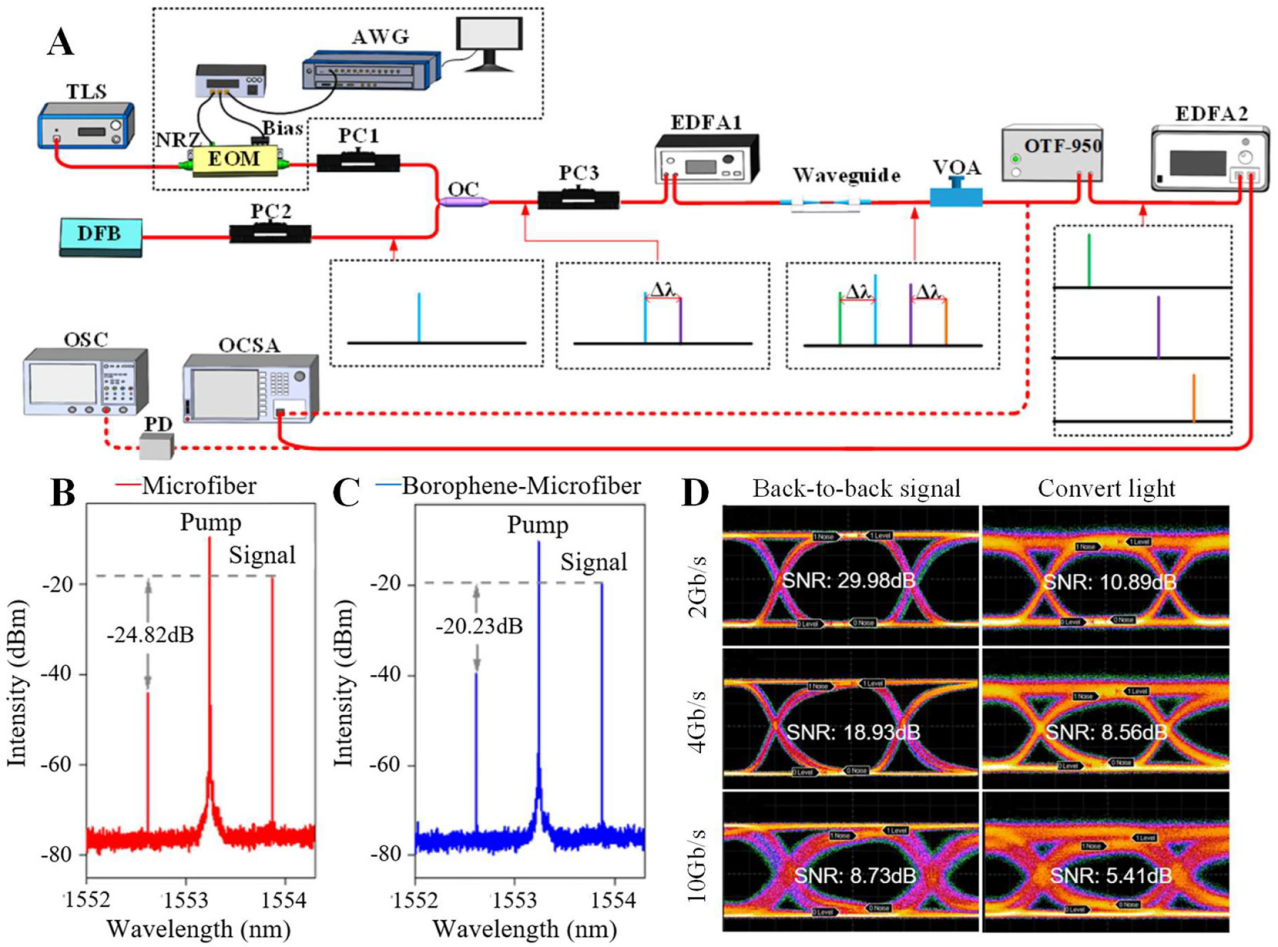
FWM wavelength conversion using Borophene-microfiber. (A) Experimental setup on FWM wavelength converter based on the Borophene-decorated microfiber. (B) Influence of the microfiber on the FWM effect. (C) Influence of the borophene-microfiber waveguide on the FWM effect. (D) Eye diagrams of back-to-back signals and converted light at modulation rates of 2, 4, and 10 Gb/s, respectively. Reproduced with permission from Ref. [156]. Copyright 2022, The Optical Society.

As a graphene-like materials, Borophene has excellent electrical, mechanical, and thermal properties, and researchers believe that Borophene will have a wider range of applications. The current research on Borophene is still in the early stage. In 2021, Ma et al. used Borophene as a saturable absorber, and proved that it has excellent optical nonlinearity in the near-infrared and mid-infrared regions, indicating that it has excellent applications in nonlinear photonic devices, etc. [154]. In 2022, Li et al. reported a Borophene-based wavelength converter for the first time [156]. The optical deposition method is used to deposit Borophene on the waist region of the microfiber, which enhances the FWM effect. Compared with the bare microfiber, the conversion efficiency in increased by about 4.6 dB. By optimizing nonlinear polarization, wavelength interval and pump power, a conversion efficiency of −19.1 dB and a 3 dB conversion bandwidth of 7.1 nm are achieved. In addition, this structure also realizes the all-optical wavelength conversion of 10 Gb/s non-return-to-zero digital sequence, as shown in Figure 18D, and further studies the signal quality of the converted light.

However, all-optical wavelength converters based on two-dimensional materials are mostly concentrated in the traditional 1.55 μm communication band. In contrast, the 2 μm band has a higher atmospheric transmittance window, but due to the need for complex processes and high-cost optical communication equipment, the construction of optical communication systems in the 2 μm waveband still faces many difficulties. For the all-optical wavelength conversion technology, due to the long distance between the 2 μm band and the zero-dispersion wavelength, and the complex structure of the wavelength converter, low conversion efficiency is the main factor limiting its application, so there are few researches on the 2 μm band [157, 158].

**Figure 19:**
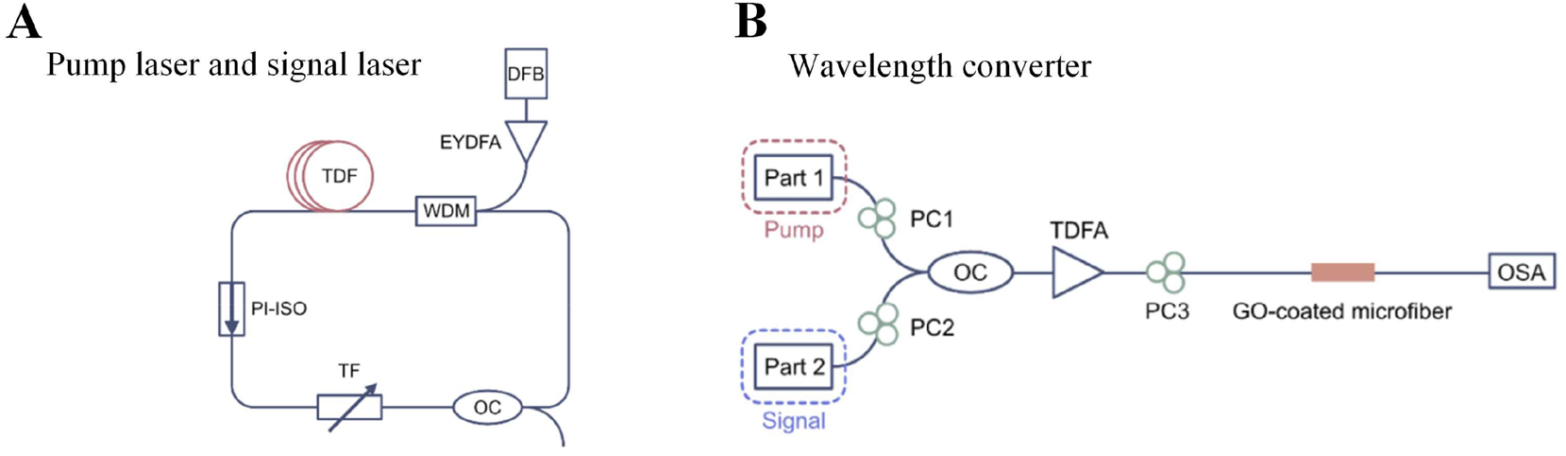
Schematic of all-optical wavelength converter based on GO. (A) The configuration of homemade thulium-doped fiber laser regarded as pump laser and signal laser. (B) The configuration of wavelength converter. Reproduced with permission from Ref. [159]. Copyright 2021, The Optical Society.

In 2021, Du et al. proposed an all-optical converter based on graphene oxide (GO) in the 1.9 μm band [159]. The experimental structure is shown in Figure 19. The self-made thulium-doped 1.9 μm laser was used as the signal laser and pump laser, self-made GO-coated microfiber with remarkable nonlinear optical response were obtained by optical deposition method. When the wavelength interval is 1 nm, the conversion efficiency can reach −45.52 dB, and the wavelength tuning range can reach 6 nm (1969∼1975 nm). In the case of a fixed wavelength interval, the conversion efficiency increases with the increase of the pump power. At the same time, the conversion efficiency fluctuation is ±0.41 dB.

**Figure 20:**
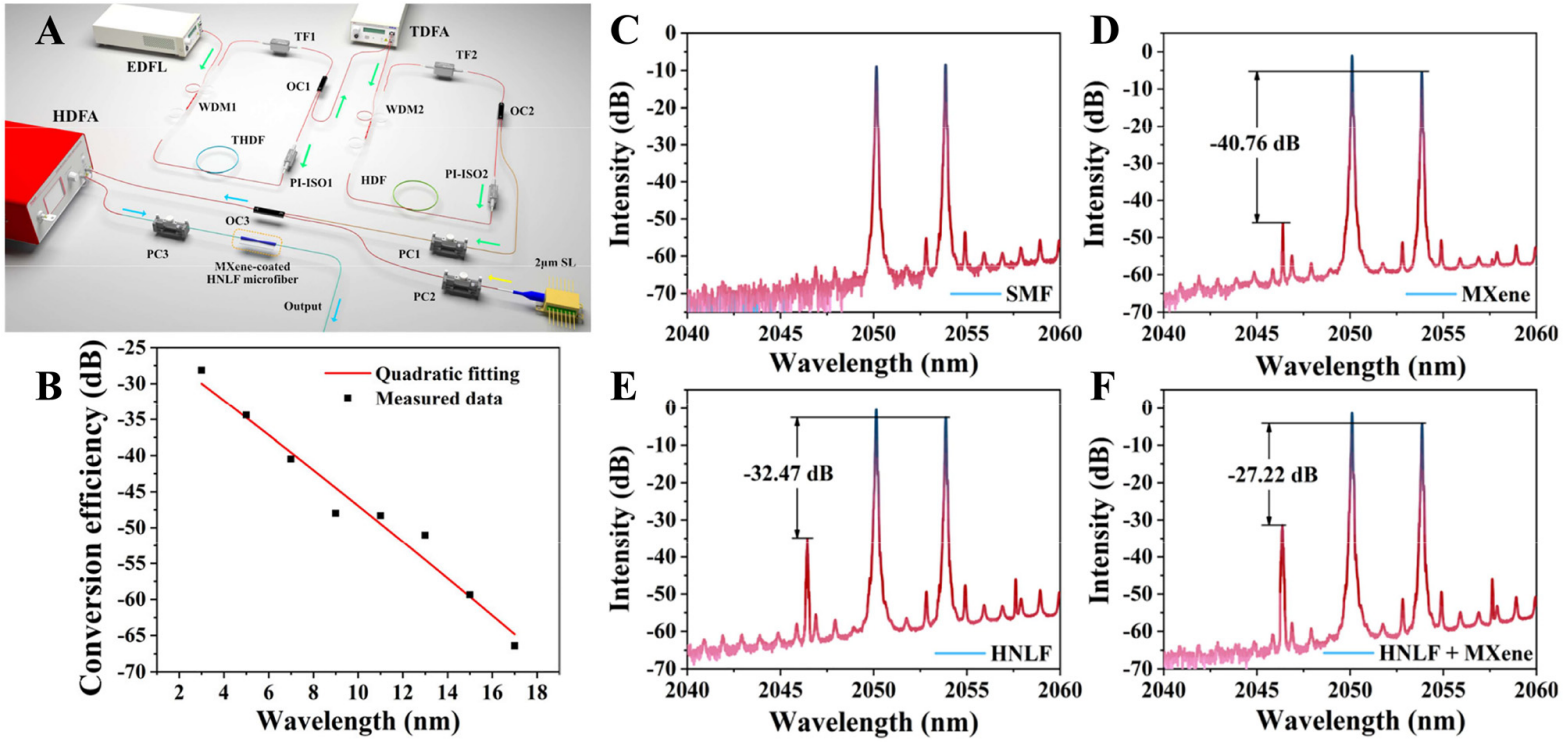
Wavelength converter based on a highly nonlinear MXene decorated microfiber. (A) Configuration of the experimental setup. (B) Magnitude of conversion efficiency against wavelength interval. (C) AOWC based on bare SMF. (D) AOWC based on tapered fiber (SMF) coated with MXene. (E) AOWC based on tapered HNLF. (F) AOWC based on tapered HNLF coated with MXene. Reproduced with permission from Ref. [160]. Copyright 2022, AIP Publishing.

In 2022, Du proposed an all-optical wavelength converter for the mid-infrared spectral range [160]. In the experiment, a self-made holmium-doped fiber laser (HDFL) was used as the pump laser, and a 2 μm semiconductor laser (SL) was used as the signal laser, and MXene was deposited on the waist region of a tapered fiber drawn from a highly nonlinear fiber by optical deposition. The experimental structure is shown in Figure 20A, the Erbium-doped Fiber Laser (EDFL) is used as the seed source of the pump laser, and the laser is output through two ring cavities. The pump laser enters the first ring cavity through a 1550/2000 nm WDM, the gain fiber adopts 3 m thulium-holmium co-doped fiber (THDF), polarization insensitive isolator (PI-ISO) ensures the unidirectional transmission of light in the ring cavity, and the function of tunable filter (TF) is to select a specific wavelength. Then, output through the 10 % port of the 10/90 coupler, and use thulium-doped fiber amplifier (TDFA) to amplify the output laser, and enter the second ring cavity through 1950/2050 nm WDM, a 3 m holmium-doped fiber (HDF) is used in the cavity, reverse pumping is used here, and TF is used to adjust the final output wavelength, output along the 20 % port of the 20/80 coupler, and the finally interact with the signal from the semiconductor laser into the microfiber to achieve wavelength conversion. It is worth noting here that when passing through the first ring cavity, the wavelength output by tunable filter is 1900 nm, because 1900 nm is the most effective wavelength absorbed by Ho^3+^, and the final output wavelength of the ring cavity is 2050.53 nm. A conversion efficiency of −27.22 dB was achieved, and the fluctuation of the conversion efficiency was kept within 1 dB within 2 h, showing good stability. At the same time, the relationship between the nonlinear medium and the conversion efficiency is also explored, as shown in Figure 20C–F. Gain range from holmium-doped fiber amplifier (HDFA) also affects conversion efficiency and wavelength spacing, the structure allows arbitrary tuning range of 17 nm. In the same year, Tao reported a single-walled carbon nanotubes (SWCNT)-based 2.05 μm all-optical wavelength converter with a conversion efficiency as high as −45.57 dB, the wavelength tuning range up to 9.72 nm [161], these studies help to realize the application of all-optical wavelength converters in 2 μm all-optical networks. Table 3 summarizes recent all-optical wavelength converters based on 2D material-integrated microfiber.

**Table 3:** All-optical wavelength converter based on different 2D materials.

Year	2D material	Length of	Diameter of	*λ* (µm)	Conversion	Ref.
		microfiber (mm)	microfiber (µm)		efficiency (dB)	
2013	CNT-polymer	100	1	1.55	−27	[162]
	Polymer	100	1	1.55	−35	[162]
2014	Graphene	70	2	1.55	−28	[148]
2015	TI: Bi_2_Te_3_	NA	5.5	1.55	−34	[133]
2015	Graphene	NA	5	1.55	−20	[149]
2017	BP	NA	7	1.55	−59.15	[151]
2018	Antimonene	NA	4.5	1.55	−63	[155]
2019	Bismuthene	NA	5	1.55	−65	[163]
2019	MXene	10	8	1.55	−59	[86]
2019	BP	NA	5	1.55	−40	[164]
2021	GO	40	8	1.9	−45.52	[159]
2022	Borophene	NA	6	1.55	−19.1	[156]
2022	MXene	45	1.1	2.05	−27.22	[160]
2022	SWCNT	30	9	2.05	−45.57	[161]

### 4.3 All-optical logic gate

In future all-optical networks, all-optical logic gates are an essential part of all-optical communication networks. The all-optical logic gate is the core device for realizing the optical switching system and the key factor for determining the performance of the network. The development of all-optical logic gates is a bridge to realize the leap from electrical computing to optical computing, which can complete the logic operation on the input binary signal at a higher rate than the electric logic gate, and it can break through the limitation of “electronic bottleneck” and improve the network capacity. However, the optical realization of logic gates is a very challenging technology. The basic idea for an all-optical logic gate is to use one signal to control the state of a binary signal carried by another beam of light. NOT, AND, OR, NAND, NOR, XOR, and XNOR are the most widely used logic gates, and they have different rules, as shown in Table 4. All-optical logic devices can be applied to node functions such as all-optical signal regeneration [165], and optical packet routing [166], and so on. Traditional all-optical logic gate devices are mainly realized by relying on nonlinear effects in highly nonlinear materials, mainly including the use of semiconductor optical amplifiers [165, 167], nonlinear optical fibers [168], micro-ring resonators [169, 170], etc. Use these as the core device to realize the all-optical logic system. At present, all-optical logic gates based on 2D material-integrated microfiber are mainly concentrated in all-optical modulators, which are briefly described here.

**Table 4:** Truth tables of common logic gates (*X* and *Y* are input binary values; *Z* is logic gate output).

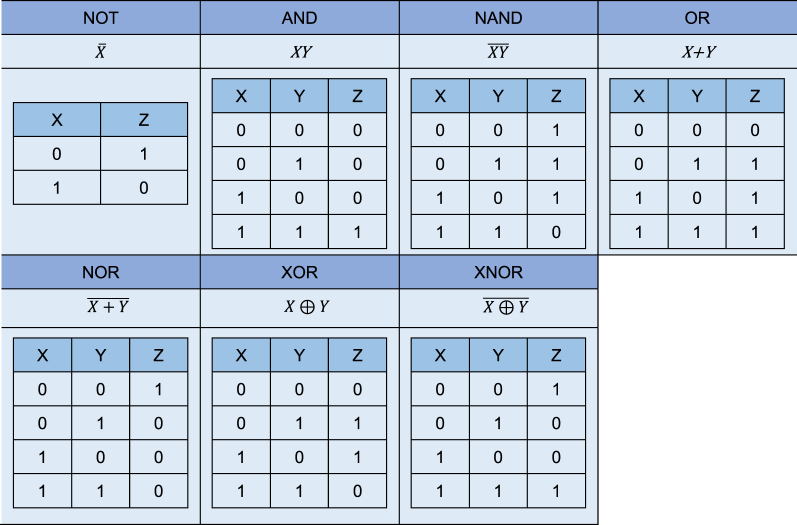

**Figure 21:**
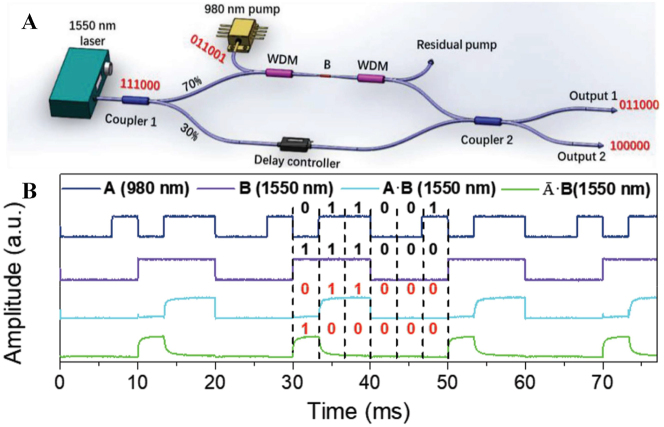
All-optical modulation based on Boron nanosheets. (A) Schematic illustration of the optical setup. (B) All-optical AND gate. Reproduced with permission from Ref. [134]. Copyright 2019, John Wiley and Sons.

In 2019, Guo et al. used liquid to peel off boron nanosheets, an all-optical modulator based on MZI structure is proposed [134]. The modulator structure can perform all-optical logic operation, the operation of the “AND” gate is verified through experiments. In the experiment, the waveforms of 980 nm (A) and 1550 nm (B) light are set to periodic “011001” and “111000” respectively. “1” and “0” represent high power and low power, respectively. When both beams are incident with high power, the output power is high, otherwise it is close to zero power output. Output 1 of the system is given by A·B (“011000”), and output 2 is given by A·B (“100000”) (as shown is Figure 21).

### 4.4 All-optical thresholding devices

In the process of optical signal transmission, due to the influence of factors such as the spontaneous emission noise of the optical amplifier and dispersion, the quality of the optical signal will gradually decrease, making the pulse quality deteriorate. Therefore, it is very important to use all-optical thresholding devices to suppress noise and improve signal-to-noise ratio. Current all-optical thresholding devices mainly utilize various nonlinear properties in optical materials. Including the nonlinear effect of special optical fiber and the second harmonic generation effect in periodically poled lithium niobate [171, 172]. All-optical thresholding devices based on two-dimensional materials mainly use the saturable absorption effect of 2D materials, the noise light with low intensity is absorbed and passes through the signal light with high intensity, so the noise with low intensity can be suppressed and the signal light can pass through with low loss, thus achieving the effect of reducing noise. Several all-optical thresholding devices based on 2D materials have been reported. For example, graphene-based all-optical thresholding devices and black phosphorus-based all-optical thresholding devices, etc. However, due to the low absorption coefficient of the graphene-based saturable absorbers, the interaction between light and substance is greatly limited, and black phosphorus is also a material that is extremely easy to oxidize in the environment [173], making its applications in all-optical thresholding devices are limited.

**Figure 22:**
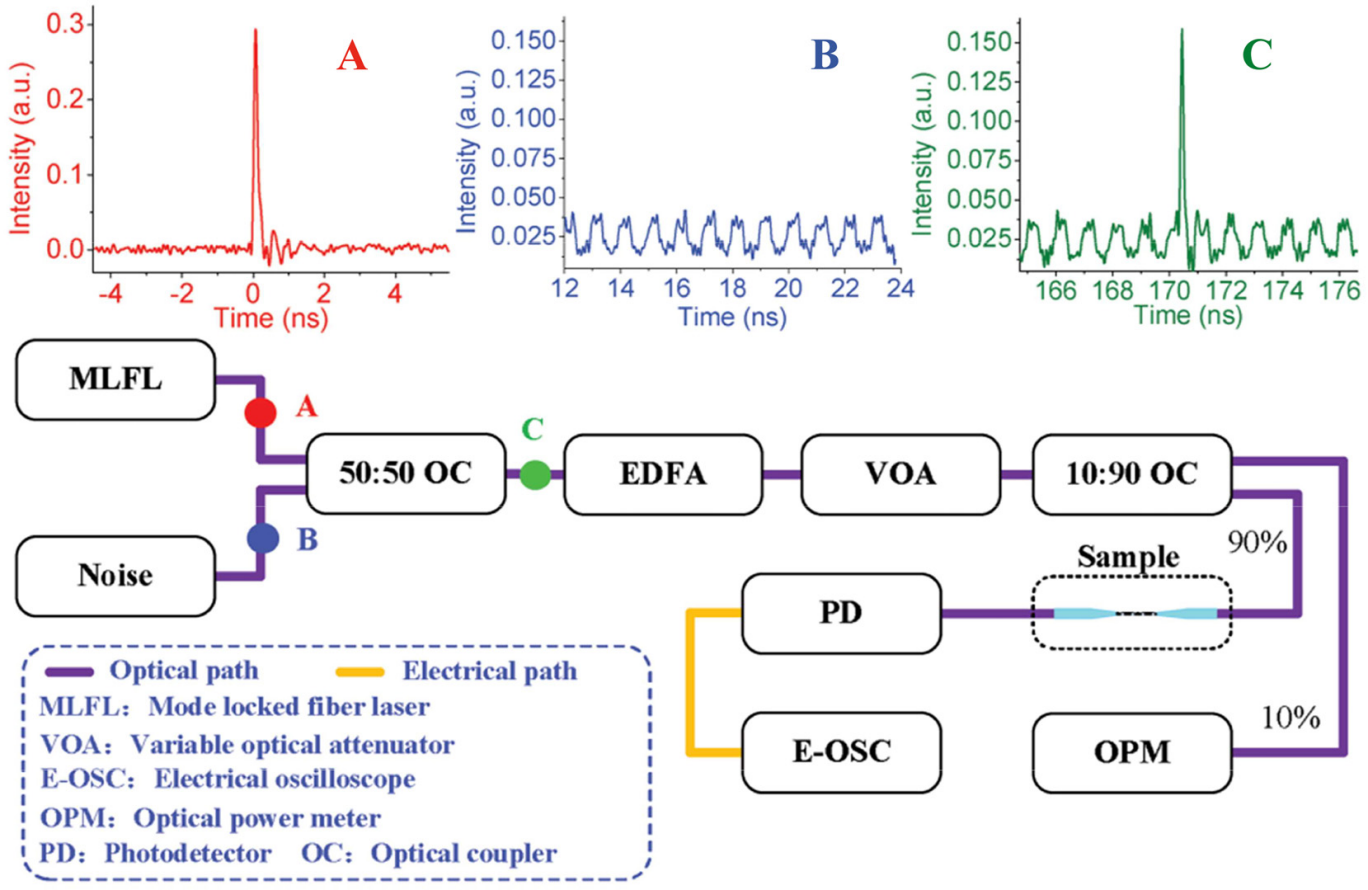
Schematic diagram of the experiment setup for all-optical thresholding with FL-P-coated microfiber. The three insets on the top are the waveforms corresponding to the three points marked with A, B, and C. Reproduced with permission from Ref. [174]. Copyright 2017, John Wiley and Sons.

**Figure 23:**
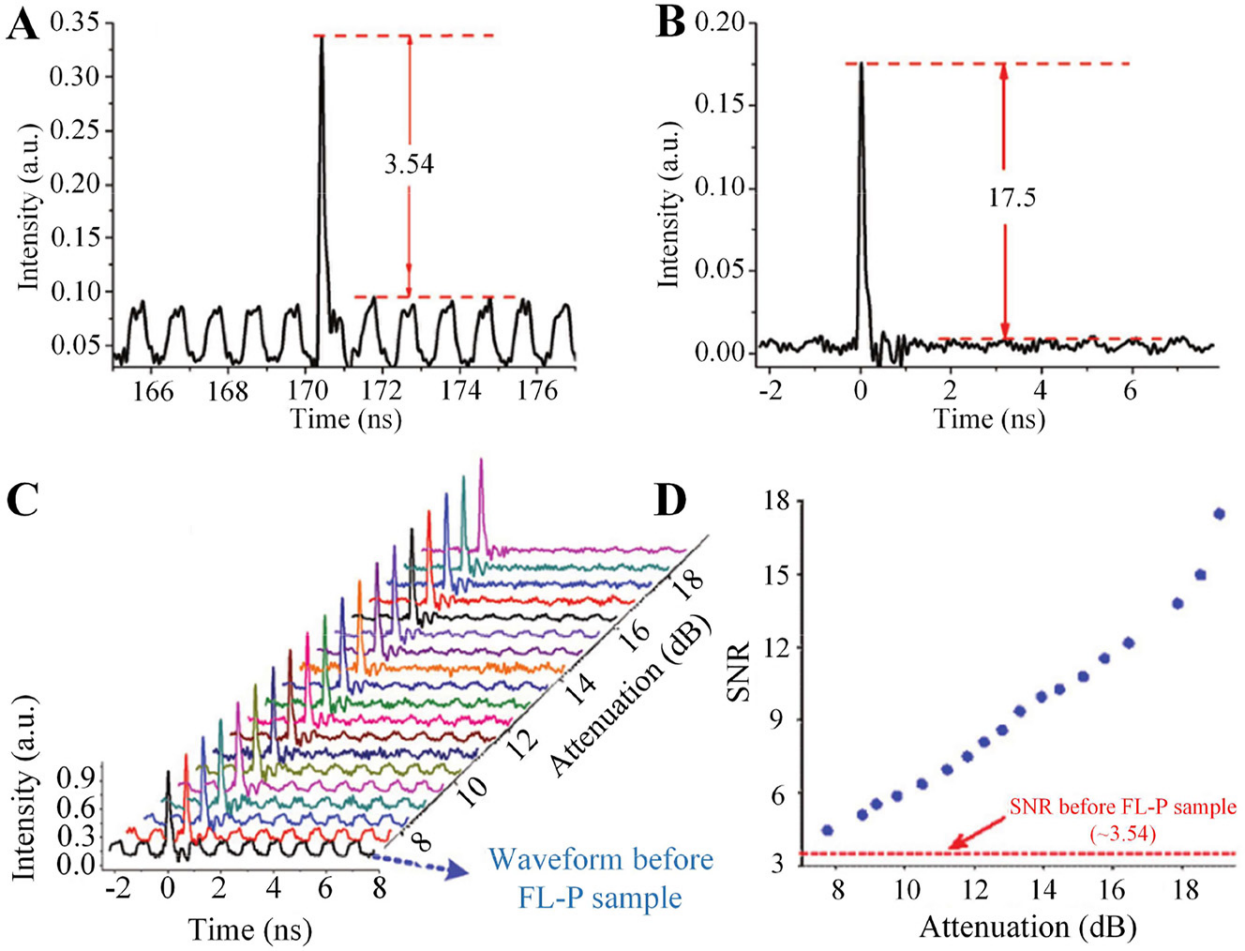
Experimental results of the all-optical thresholding. (A) The optical pulse waveform before the sample. (B) The optical pulse waveform after the sample. (C) The entire evolution process of pulse waveforms when the VOA is turned from 7.75 to 19.03 dB while the output of EDFA is kept at 22.25 dBm. (D) The corresponding SNR against the value of VOA. Reproduced with permission from Ref. [174]. Copyright 2017, John Wiley and Sons.

In 2017, Zheng et al. used the direct band gap and resonant absorption properties of four-layer phosphorus in the communication band, few-layer phosphorus (FL-P) deposition onto microfiber for all-optical thresholding [174]. FL-P (mainly four layers) was fabricated by electrochemical cathodic stripping combined with centrifugation technology, which can exclude potential oxidation conditions. The diagram of the all-optical thresholding experimental device is shown in Figure 22, a Mode locked fiber laser (MLFL) is used as the signal light, and low-power light modulated by radio frequency modulator is used to simulate noise light. After the noise is coupled with pulsed light, the mixed light is divided into two optical paths through the optical amplifier and adjustable attenuation, one path is connected to the optical power meter, and the other path is processed through the microfiber for thresholding. The waveform diagrams of points A, B and C in the device correspond to Figure 22A–C. After passing through the all-optical thresholding device, the signal-to-noise ratio of the pulse laser is significantly improved, and the signal-to-noise ratio is increased from 3.54 dB to 17.5 dB (as shown in Figure 23A and B), and the noise interference in the pulse signal is suppressed. In order to further explore the noise suppression effect under different input optical powers, the output power pf the EDFA is controlled to 22.25 dB, and the Variable Optical Attenuator (VOA) is turned from 7.75 dB to 19.03 dB. The process of pulse evolution and the relationship curve between attenuation and SNR is shown in Figure 23D. It can be seen that with the increase of attenuation, the signal-noise ratio of the pulse signal will also increase, this is mainly due to the fact that when the input power is much higher than the saturation power, both signal laser and noise can penetrate the sample proportionally, and noise suppression plays a secondary role; when the input power is close to or even lower than the saturation power, the transmittance of signal laser is higher than that of noise, and noise suppression plays a major role. At the same time, it should be avoided to keep the input power at a low level, because when the incident power is low, the detector will generate unavoidable noise. By properly controlling the input power, a better all-optical thresholding function can be achieved, the received signal quality can be improved, and the performance of the communication system can be further improved.

In 2018, Ge et al. deposited TiS_2_ on a microfiber as an all-optical thresholding device [175], they verified by experiments that the modulation depth and saturation intensity of TiS_2_ were higher than those of graphene [22]. In the experiment, a pulse source with a center wavelength of 1566 nm, a pulse width of about 400 fs, and a repetition rate of 20 Mhz was used as the signal light, and a light source with a center wavelength of 1550 nm modulated by 1 Ghz radio frequency modulator was used as the noise source. Finally, in order to verify the performance of the thresholding device, it was compared with the bare microfiber, and the stability test was carried out at the same time. The experimental results show that the thresholding device can effectively attenuate the noise and increase the signal-to-noise ratio from 1.90 dB to 10.68 dB. Compared with the bare microfiber, the device has a stronger ability to suppress noise, and the device also has good stability. Using this scheme, all-optical thresholding devices based on microfiber devices modified by other 2D materials have also been reported [176–178] (Table 5).

**Table 5:** Comparison of all-optical thresholding devices based on different 2D materials.

Year	2D material	Deposition	Diameter of	SNR (dB)	Ref.
		length (µm)	microfiber (µm)		
2017	Phosphorene	176	4.4	3.54–17.5	[174]
2017	Antimonene	240	8	1.902–11.89	[176]
2018	TiS_2_	185	6	1.90–10.68	[175]
2019	NiS_2_	NA	3	2.7–14.5	[177]
2019	PbO	190	8	2.81–8.01	[178]

## 5 Conclusions and future perspectives

Two-dimensional materials, represented by graphene and black phosphorus, have attracted great attention from researchers in recent years due to their excellent optical, electrical, magnetic, and force properties, and in-depth research has been conducted on the properties and preparation of emerging 2D materials. The continuous maturation of the preparation process will simplify the synthesis of 2D materials and further reduce the production cost. Emerging 2D materials have also been discovered and have shown great potential for their application in optoelectronic devices. The excellent performance and good integration ability of 2D materials have shown that it will become a favorable tool to improve the performance of traditional optical waveguides. Due to the characteristics of all-fiber structure, high nonlinearity and high damage threshold, the two-dimensional materials-integrated microfiber composite waveguide structure has been widely used in photonics, nano-devices, and other fields, and has achieved many excellent results. The development of optoelectronic devices cannot be solved by the development of 2D materials alone, but their discovery has made an important contribution to their development into commercial devices. This paper reviews the recent progress in the application of two-dimensional material-integrated microfiber composite waveguides in all-optical signal processing technology. First, the optoelectronic properties of some typical two-dimensional materials and common methods of fabricating microfibers and two-dimensional materials are introduced, and the methods commonly used in the laboratory for fabrication two-dimensional materials integrated with microfiber composite waveguides are presented. Then, in the fourth part, the applications of composite waveguides in all-optical modulators, all-optical wavelength converters, all-optical logic gates and all-optical thresholding devices are elaborated. The main advantage of composite waveguides applied to optoelectronic devices through the interaction of two-dimensional materials with light, and the saturable absorption effect, thermo-optic effect and nonlinear effect of two-dimensional materials are mainly utilized in all-optical signal processing techniques.

For all-optical modulators, compared with modulators with MZI and MI structures, the MKR structure can reduce the volume of two-dimensional materials and improve the overall transmittance of the system, and have better performance in response time, which will be an important direction for the development of all-optical modulators in the future. In addition, the thermo-optic effect possessed by two-dimensional materials makes its application in phase shifters and optical switches more and more mature, but it is easily affected by the environment, and the thermo-optic effect will also interfere with the modulation effect of other effects. At the same time, wavelength converters and thresholding devices based on 2D materials also have higher requirements due to their third-order nonlinearity and saturable absorption effects. Therefore, the exploration of new 2D materials is still a hot topic at present.

Although the application capability of 2D material-integrated microfiber composite waveguide in all-optical signal processing technology has been verified by experiments, it is still limited to laboratory research, and commercialization and industrialization still face great challenges. For example, the composite waveguide structure is very fragile, very sensitive to the environment, it needs to be protected; secondly, the preparation process of the composite waveguide is difficult to control, resulting in very unstable parameters, which limits its actual production; the insertion loss of the composite waveguide is large; the photothermal effect caused by the interaction between light and materials in practical applications. It will take a long time to overcome these difficulties.

Based on the above challenges, future perspectives are proposed. First, the main advantage of the two-dimensional material-integrated microfiber composite waveguide is its tiny structure. How to improve its stability and how to make it into an integrated device that can be used stably is the key direction of future research. Second, the application of composite waveguides in the deep ultraviolet and mid-infrared wavebands is very limited, so there are strong requirements for the development of composite waveguides in the mid-infrared and deep ultraviolet wavebands. Then, due to the different parameters of various 2D materials, the required properties in specific cases are also different. It is necessary to systematically study the unique properties of 2D materials in order to select 2D materials and parameters in specific areas. Finally, with the continuous maturation of the preparation process, it is believed that more and more new 2D materials will be discovered, and changing their structures by specific methods will diversify their functions, studying their nonlinear properties and developing related devices will be an important research direction in the future. It is believed that the 2D material-integrated microfiber composite waveguide will continue to develop rapidly, realize commercialization and practical application as soon as possible, and open new doors in new fields.
